# Supplementary material for: Engineering a transposon-associated TnpB-ωRNA system for efficient gene editing and phenotypic correction of a tyrosinaemia mouse model
Source: Nat Commun. 2024 Jan 27;15:831. doi: 10.1038/s41467-024-45197-z (PMC10821889; doi:10.1038/s41467-024-45197-z)
Supplement: Supplementary file 1 — Supplementary Information [file 41467_2024_45197_MOESM1_ESM.pdf]

Supplementary materials for

Title

Engineering a transposon-associated TnpB-ωRNA system for efficient gene editing and phenotypic correction of a tyrosinaemia mouse model

Authors

Zhifang Li[1†], Ruochen Guo[1,6†], Xiaozhi Sun[1,2†], Guoling Li[4†], Zhuang Shao[1], Xiaona Huo[1,2], Rongrong Yang[1,2], Xinyu Liu[6], Xi Cao[1,9], Hainan Zhang[4], Weihong Zhang[4], Xiaoyin Zhang[1,2], Shuangyu Ma[7], Meiling Zhang[8], Yuanhua Liu[6], Yinan Yao[6], Jinqi Shi[1], Hui Yang[3,4,6], Chunyi Hu[5*], Yingsi Zhou[4*], Chunlong Xu[1,2,3*]

[†]These authors contributed equally to this work.

***Correspondences**

hu_dbs@nus.edu.sg (C.H.), yingsizhou@huidagene.com (Y.Z.), xucl@lglab.ac.cn (C.X.)

This PDF file includes

Fig. S1. Deep-seq reads analysis results for *Tyr* gene-edited mice.

Fig. S2. Deep-seq reads analysis results for *Dmd* gene-edited mice.

Fig. S3. RNA genotyping of *Dmd*-edited mice with reverse transcription and targeted PCR by sequencing.

Fig. S4. Dystrophin and laminin-2 immunostaining results for TA, DI and heart muscle in *Dmd*-edited mice.

Fig. S5. Grip strength and rotarod test for *Dmd*-edited mice.

Fig. S6. Gene editing activity comparison among TnpB-ωRNA, TnpB-ωRNA*, SaCas9 and SpCas9 in N2a (a) and HEK293T cells (b,c).

Fig. S7. Deep-seq reads analysis results for site1 edited with TnpB-ωRNA/ωRNA* in HEK293T cells.

Fig. S8. Deep-seq reads analysis results for *Hpd* edited with TnpB-

ωRNA/ωRNA* in N2a cells.
Fig. S9. Deep-seq reads analysis results for *Hpd* edited with SaCas9/SpCas9 in N2a cells.
Fig. S10. Characterization of gene editing activity for engineered TnpB-ωRNA system in mouse N2a cells.
Fig. S11. *In vivo* characterization of gene editing activity for the engineered TnpB-ωRNA* system delivered by AAV in mice.
Fig. S12. Comparison of therapeutic efficacy between TnpB and Un1Cas12f1 in HT1 mice.
Fig. S13. Immunostaining and gene editing analysis for HPD in AAV-TnpB-ωRNA treated mouse liver.
Fig. S14. Plasma and urine biochemical analysis for AAV-TnpB-ωRNA treated mouse liver.
Fig. S15. TnpB expression in E. coli. and in vitro DNA cleavage analysis.
Fig. S16. Antibody analysis in the human blood for TnpB and SpCas9.
Fig. S17. Figure exemplifying the gating strategy for Flow Cytometry.
Table S1. Gene editing efficiency for TnpB targeting human 14 sites.
Table S2. Target sgRNA and primer sequence.
Table S3. PCR and IVT primers used in this study.
Table S4. NGS primers used in this study.
Table S5. Human 14 target sites sequences and oligos for TnpB activity evaluation in this study.
Table S6. SaCas9 target site sequences in this study.
Table S7. SpCas9 target site sequences in this study.
Supplementary sequences

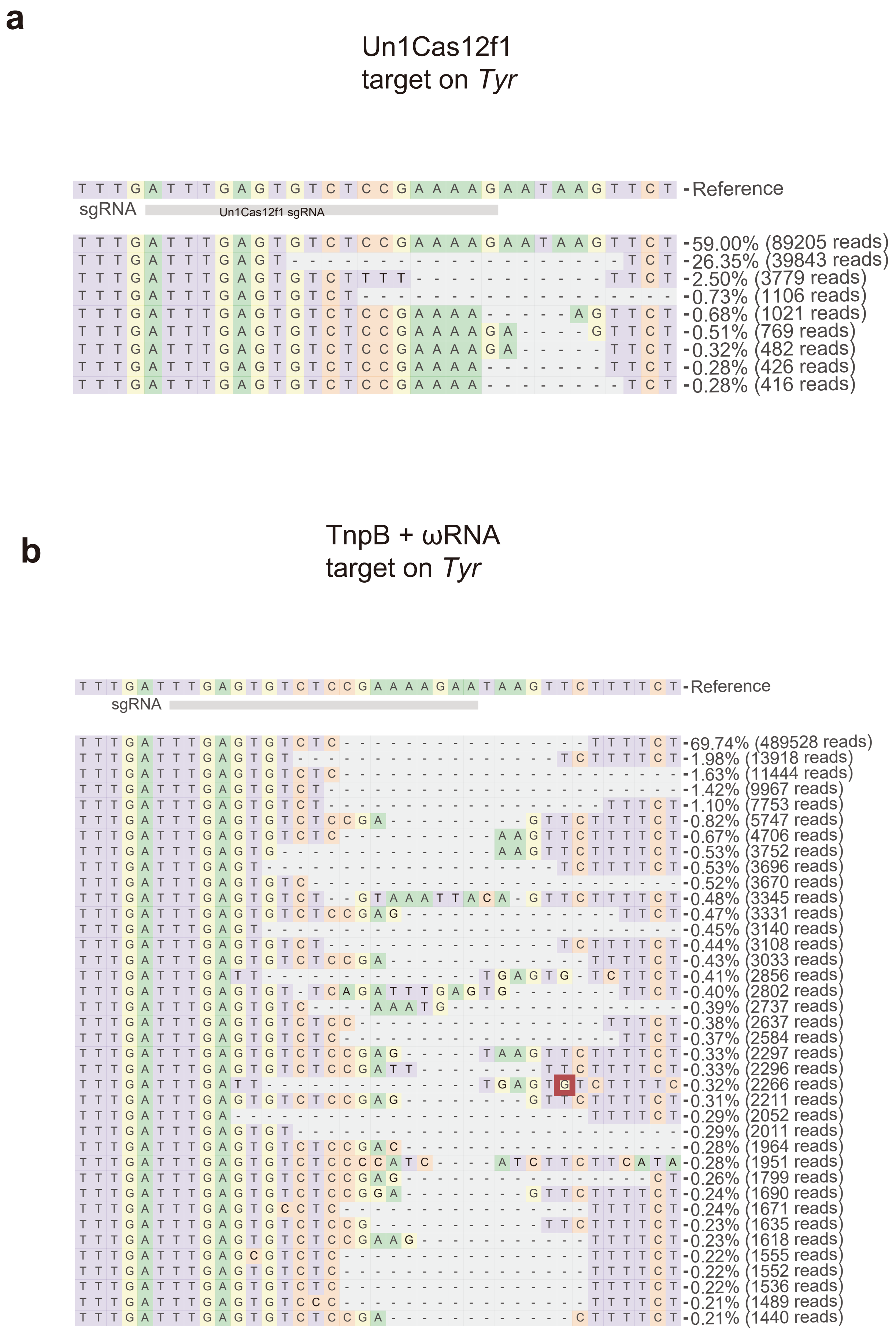
a
Un1Cas12f1
target on *Tyr*
Reference
sgRNA
Un1Cas12f1 sgRNA
59.00% (89205 reads)
26.35% (39843 reads)
2.50% (3779 reads)
0.73% (1106 reads)
0.68% (1021 reads)
0.51% (769 reads)
0.32% (482 reads)
0.28% (426 reads)
0.28% (416 reads)
b
TnpB + ωRNA
target on *Tyr*
Reference
sgRNA
69.74% (489528 reads)
1.98% (13918 reads)
1.63% (11444 reads)
1.42% (9967 reads)
1.10% (7753 reads)
0.82% (5747 reads)
0.67% (4706 reads)
0.53% (3752 reads)
0.53% (3696 reads)
0.52% (3670 reads)
0.48% (3345 reads)
0.47% (3331 reads)
0.45% (3140 reads)
0.44% (3108 reads)
0.43% (3033 reads)
0.41% (2856 reads)
0.40% (2802 reads)
0.39% (2737 reads)
0.38% (2637 reads)
0.37% (2584 reads)
0.33% (2297 reads)
0.33% (2296 reads)
0.32% (2266 reads)
0.31% (2211 reads)
0.29% (2052 reads)
0.29% (2011 reads)
0.28% (1964 reads)
0.28% (1951 reads)
0.26% (1799 reads)
0.24% (1690 reads)
0.24% (1671 reads)
0.23% (1635 reads)
0.23% (1618 reads)
0.22% (1555 reads)
0.22% (1552 reads)
0.22% (1536 reads)
0.21% (1489 reads)
0.21% (1440 reads)

Fig.S1

Supplementary figures and legend

Fig. S1. Deep-seq reads analysis results for *Tyr* gene-edited mice.

a. Deep-seq reads analysis results for *Tyr* gene-edited mice by Un1Cas12f1.

b. Deep-seq reads analysis results for *Tyr* gene-edited mice by TnpB.

TnpB + ωRNA
target on *Dmd*

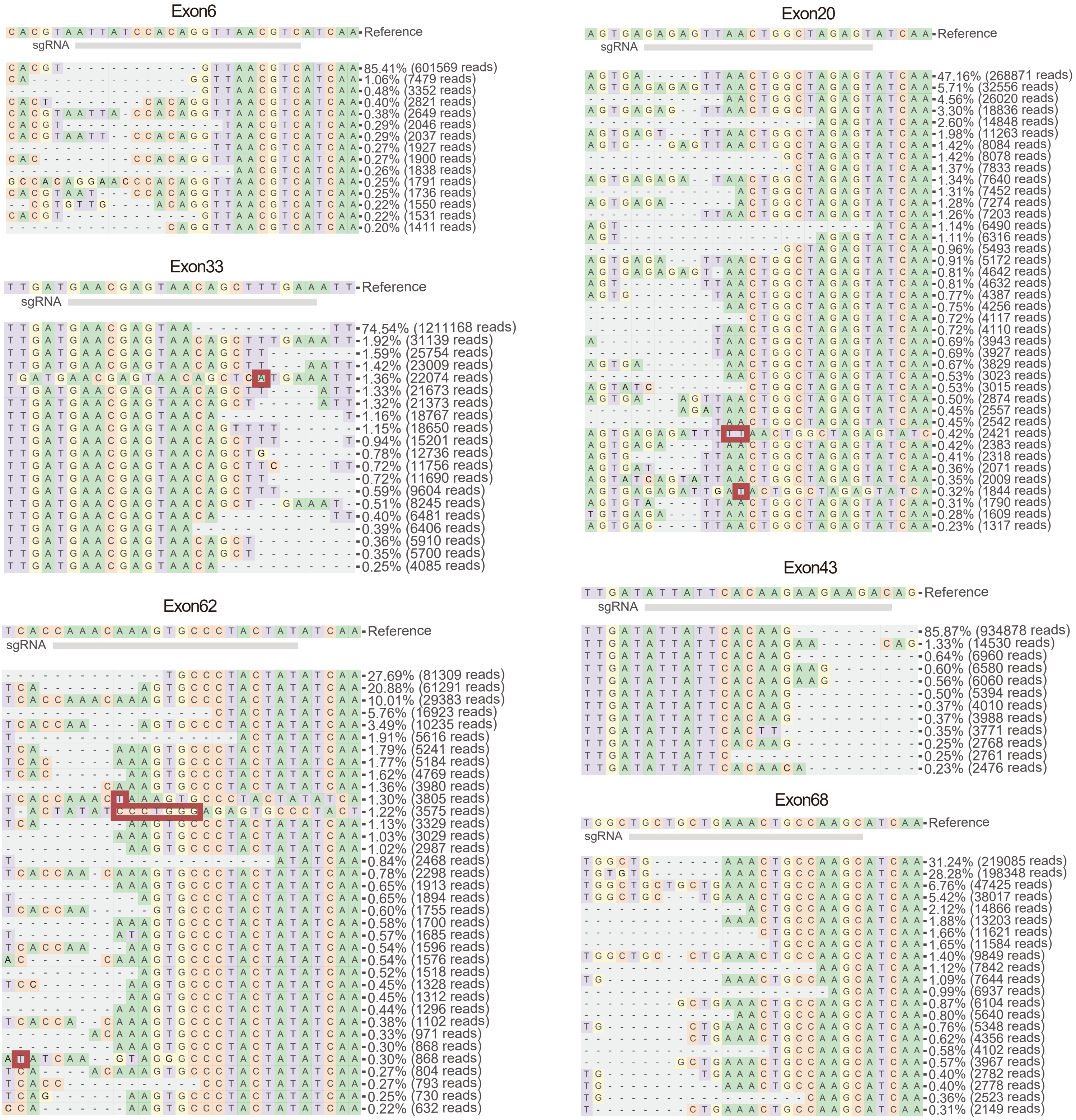

Fig.S2

Fig. S2. Deep-seq reads analysis results for *Dmd* gene-edited mice.

Deep-seq reads analysis results for *Dmd* gene-edited mice from individual mouse edited by TnpB in exon 6, 20, 33, 43, 62 and 68 of *Dmd* gene.

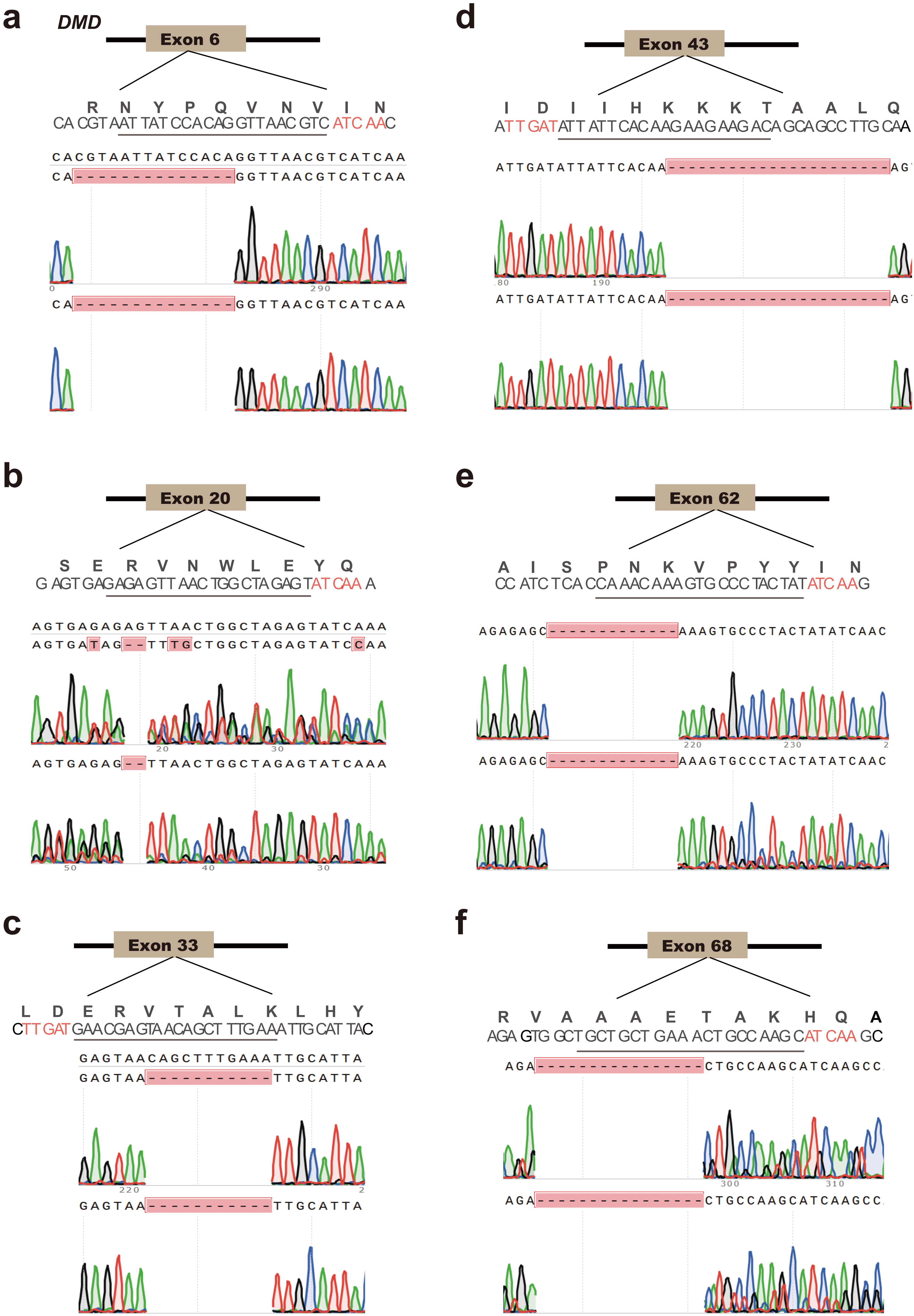
a
DMD
Exon 6
R N Y P Q V N V I N
CA CGT AAT TAT CCA CAG GTT AAC GTC ATC AAC
CACGTAATTATCCACAGGTTAACGTCATCAA
CA--------------GGTTAACGTCATCAA
CA--------------GGTTAACGTCATCAA
b
Exon 20
S E R V N W L E Y Q
G AGT GAG AGA GTT AAC TGG CTA GAG TAT CAA A
AGTGAGAGAGTTAACTGGCTAGAGTATCAAA
AGTGATAG--TTTGCTGGCTAGAGTATCCAA
AGTGAGAG--TTAACTGGCTAGAGTATCAAA
c
Exon 33
L D E R V T A L K L H Y
CTT GAT GAA CGA GTA ACA GCT TTG AAA TTG CAT TAC
GAGTAACAGCTTTGAAATTGCATTA
GAGTAA-----------TTGCATTA
GAGTAA-----------TTGCATTA
d
Exon 43
I D I I H K K K T A A L Q
ATT GAT ATT ATT CAC AAG AAG AAG ACA GCA GCC TTG CAA
ATTGATATTATTCACAA--------------------AG
ATTGATATTATTCACAA--------------------AG
e
Exon 62
A I S P N K V P Y Y I N
CC ATC TCA CCA AAC AAA GTG CCC TAC TAT ATC AAG
AGAGAGC------------AAAGTGCCCTACTATATCAAC
AGAGAGC------------AAAGTGCCCTACTATATCAAC
f
Exon 68
R V A A A E T A K H Q A
AGA GTG GCT GCT GCT GAA ACT GCC AAG CAT CAA GC
AGA---------------CTGCCAAGCATCAAGCC
AGA---------------CTGCCAAGCATCAAGCC

Fig.S3

Fig. S3. RNA genotyping of *Dmd*-edited mice with reverse transcription and targeted PCR by sequencing.

a-f. RNA genotyping results after reverse transcription and targeted PCR (RT-PCR) by sequencing for muscle from individual mouse edited by TnpB in exon 6, 20, 33, 43, 62 and 68 of *Dmd* gene.

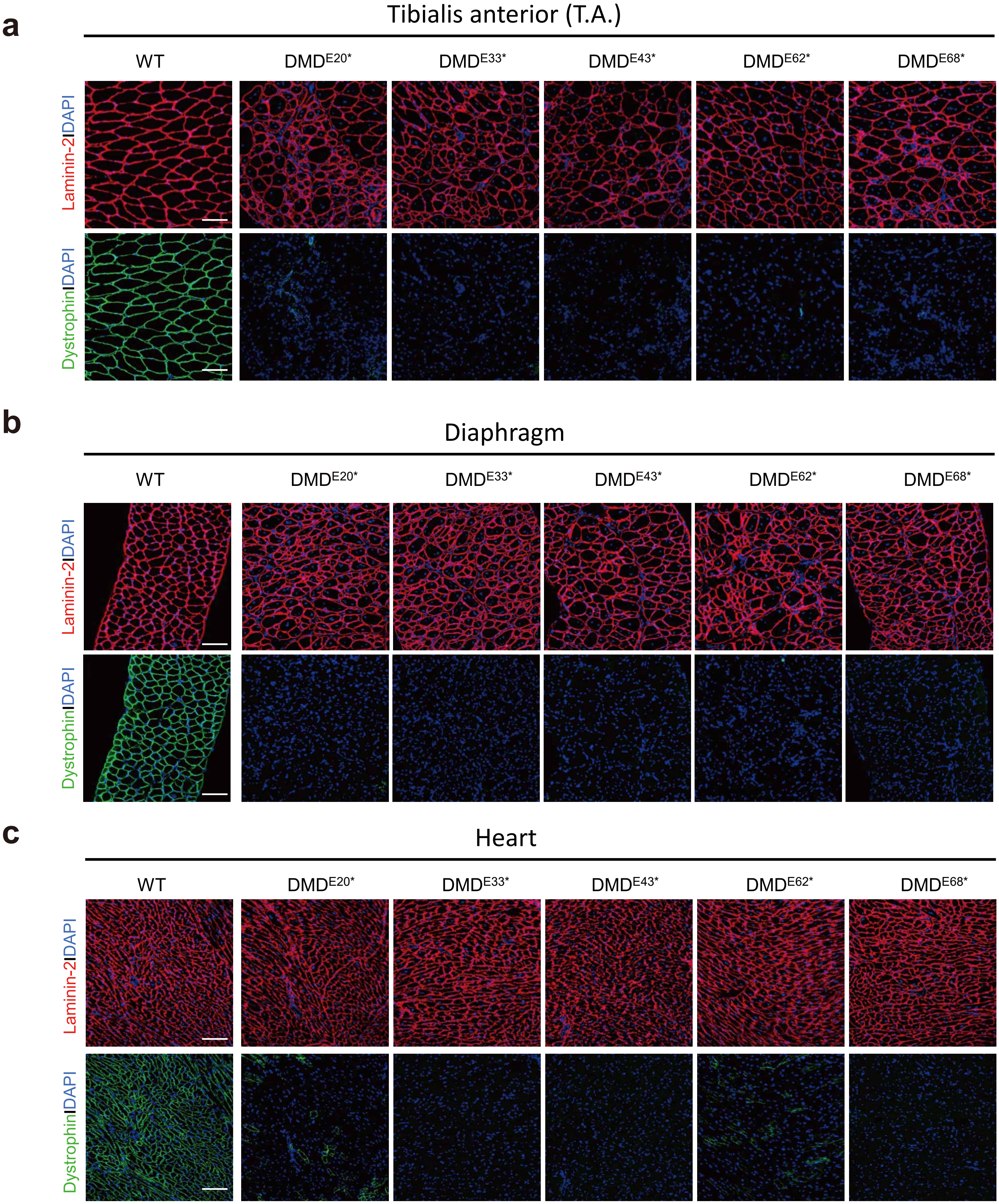
a
Tibialis anterior (T.A.)
WT
DMDE20*
DMDE33*
DMDE43*
DMDE62*
DMDE68*
Laminin-2IDAPI
DystrophinIDAPI
b
Diaphragm
WT
DMDE20*
DMDE33*
DMDE43*
DMDE62*
DMDE68*
Laminin-2IDAPI
DystrophinIDAPI
c
Heart
WT
DMDE20*
DMDE33*
DMDE43*
DMDE62*
DMDE68*
Laminin-2IDAPI
DystrophinIDAPI

Fig.S4

Fig. S4. Dystrophin and laminin-2 immunostaining results for TA, DI and heart muscle in *Dmd*-edited mice.

a-c. Immunostaining of dystrophin and laminin-2 in TA, DI and heart muscle from mice edited by TnpB in exon 6, 20, 33, 43, 62 and 68 of *Dmd* gene. Scale bars, 100 μm.

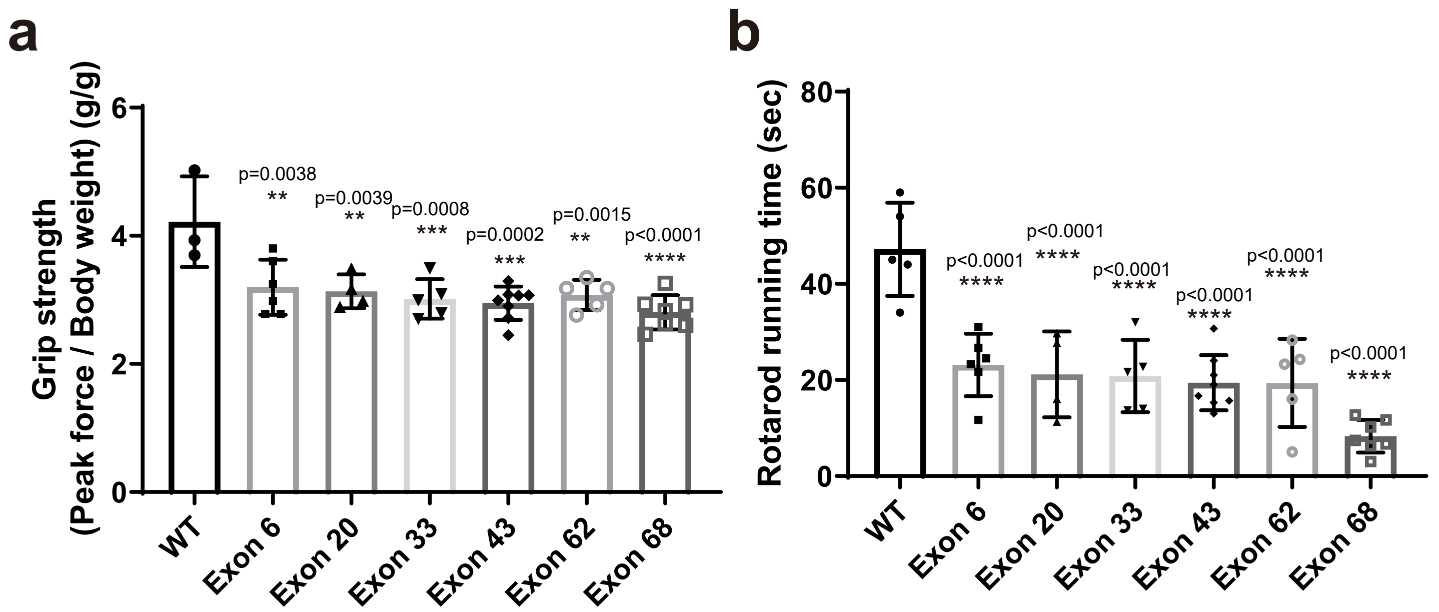
a
Grip strength
(Peak force / Body weight) (g/g)
0
2
4
6
p=0.0038
**
p=0.0039
**
p=0.0008
***
p=0.0002
***
p=0.0015
**
p<0.0001
****
WT
Exon 6
Exon 20
Exon 33
Exon 43
Exon 62
Exon 68
b
Rotarod running time (sec)
0
20
40
60
80
p<0.0001
****
p<0.0001
****
p<0.0001
****
p<0.0001
****
p<0.0001
****
p<0.0001
****
WT
Exon 6
Exon 20
Exon 33
Exon 43
Exon 62
Exon 68

Fig.S5

Fig. S5. Grip strength and rotarod test for *Dmd*-edited mice.

a. Forelimb grip strength analysis results for wildtype and *Dmd* mutant mice.

b. Rotarod running time analysis results for wildtype and *Dmd* mutant mice.

Data are represented as means ± SEM. A dot represents a biological replicate. Significant differences between conditions are indicated by asterisk. A dot represents a biological replicate (n=3 or more). One-way ANOVA analysis. Significant differences between conditions are indicated by asterisk (* $P < 0.05$, ** $P<0.01$, *** $P < 0.001$, **** $P < 0.0001$, NS non-significant.). Source data are provided in the Source Data File.

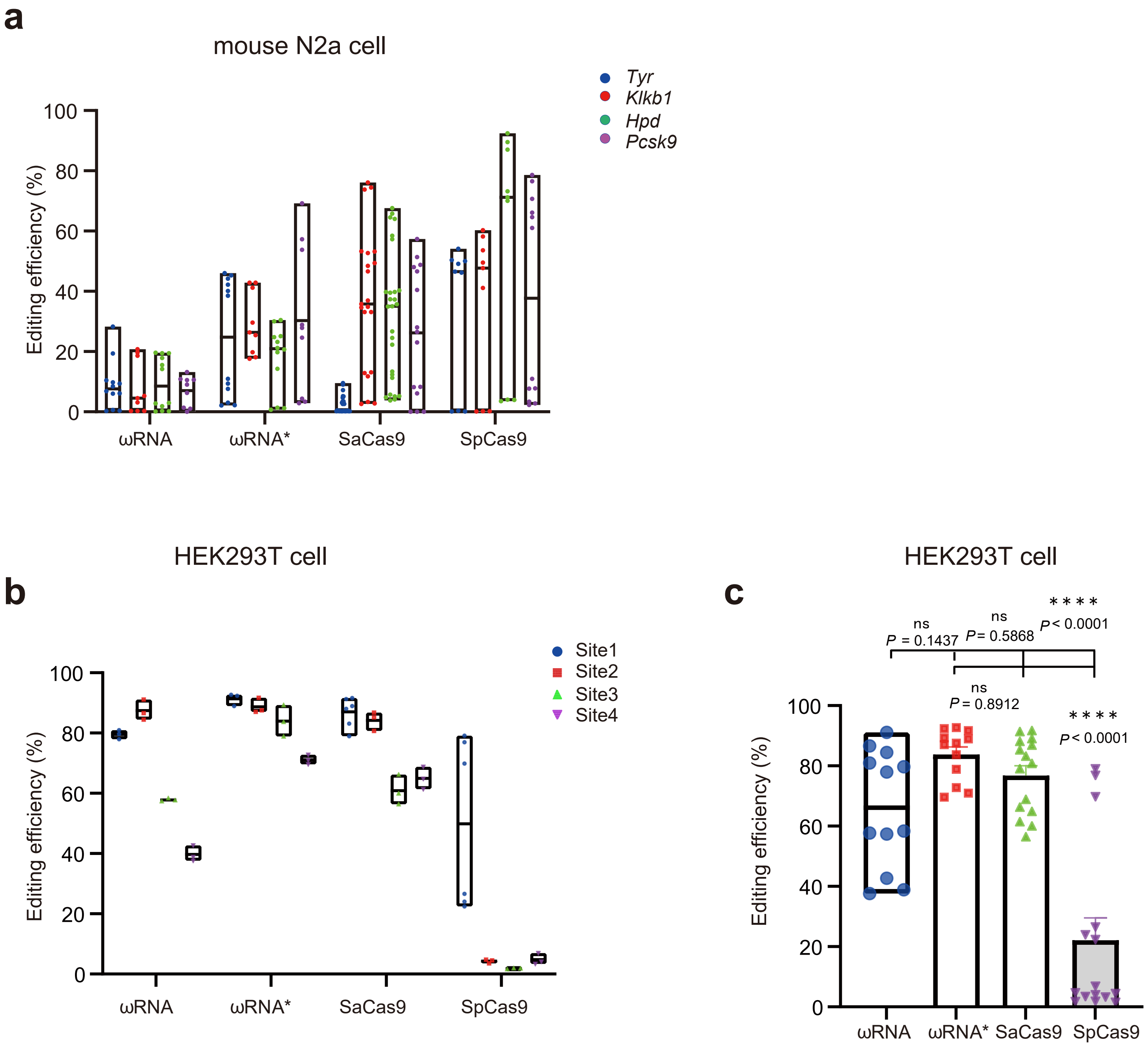
a
mouse N2a cell
Tyr
Klkb1
Hpd
Pcsk9
Editing efficiency (%)
100
80
60
40
20
0
ωRNA
ωRNA*
SaCas9
SpCas9
b
HEK293T cell
Site1
Site2
Site3
Site4
Editing efficiency (%)
ωRNA
ωRNA*
SaCas9
SpCas9
c
HEK293T cell
ns
P = 0.1437
ns
P = 0.5868
****
P < 0.0001
ns
P = 0.8912
****
P < 0.0001
Editing efficiency (%)
ωRNA
ωRNA*
SaCas9
SpCas9

Fig.S6

Fig. S6. Gene editing activity comparison among TnpB-ωRNA, TnpB-ωRNA*, SaCas9 and SpCas9 in N2a (a) and HEK293T cells (b, c). A dot represents a biological replicate (n=3 or more). Unpaired two-tailed Student's t tests. * P < 0.05, ** P<0.01, *** P < 0.001, **** P < 0.0001, NS non-significant. Source data are provided in the Source Data File.

a

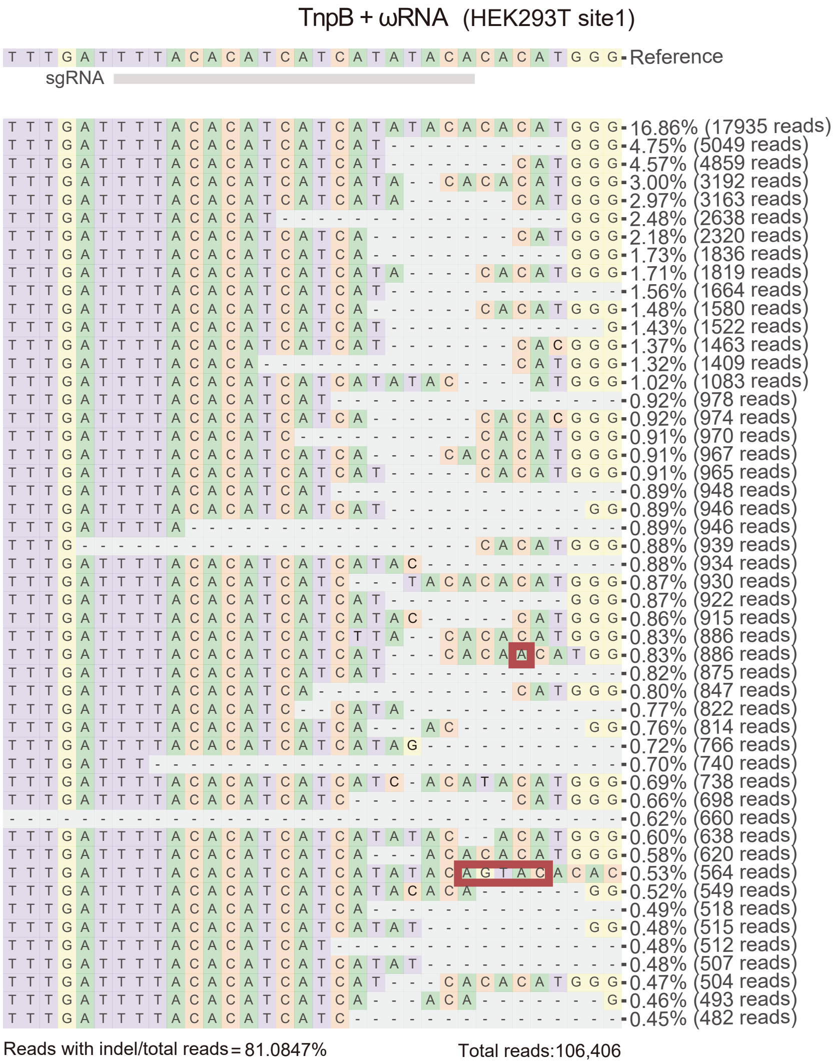
TnpB + ωRNA (HEK293T site1)
TTTGATTTTACACATCATCATATACACACATGGG Reference
sgRNA
16.86% (17935 reads)
4.75% (5049 reads)
4.57% (4859 reads)
3.00% (3192 reads)
2.97% (3163 reads)
2.48% (2638 reads)
2.18% (2320 reads)
1.73% (1836 reads)
1.71% (1819 reads)
1.56% (1664 reads)
1.48% (1580 reads)
1.43% (1522 reads)
1.37% (1463 reads)
1.32% (1409 reads)
1.02% (1083 reads)
0.92% (978 reads)
0.92% (974 reads)
0.91% (970 reads)
0.91% (967 reads)
0.91% (965 reads)
0.89% (948 reads)
0.89% (946 reads)
0.89% (946 reads)
0.88% (939 reads)
0.88% (934 reads)
0.87% (930 reads)
0.87% (922 reads)
0.86% (915 reads)
0.83% (886 reads)
0.83% (886 reads)
0.82% (875 reads)
0.80% (847 reads)
0.77% (822 reads)
0.76% (814 reads)
0.72% (766 reads)
0.70% (740 reads)
0.69% (738 reads)
0.66% (698 reads)
0.62% (660 reads)
0.60% (638 reads)
0.58% (620 reads)
0.53% (564 reads)
0.52% (549 reads)
0.49% (518 reads)
0.48% (515 reads)
0.48% (512 reads)
0.48% (507 reads)
0.47% (504 reads)
0.46% (493 reads)
0.45% (482 reads)
Reads with indel/total reads = 81.0847%
Total reads:106,406

b

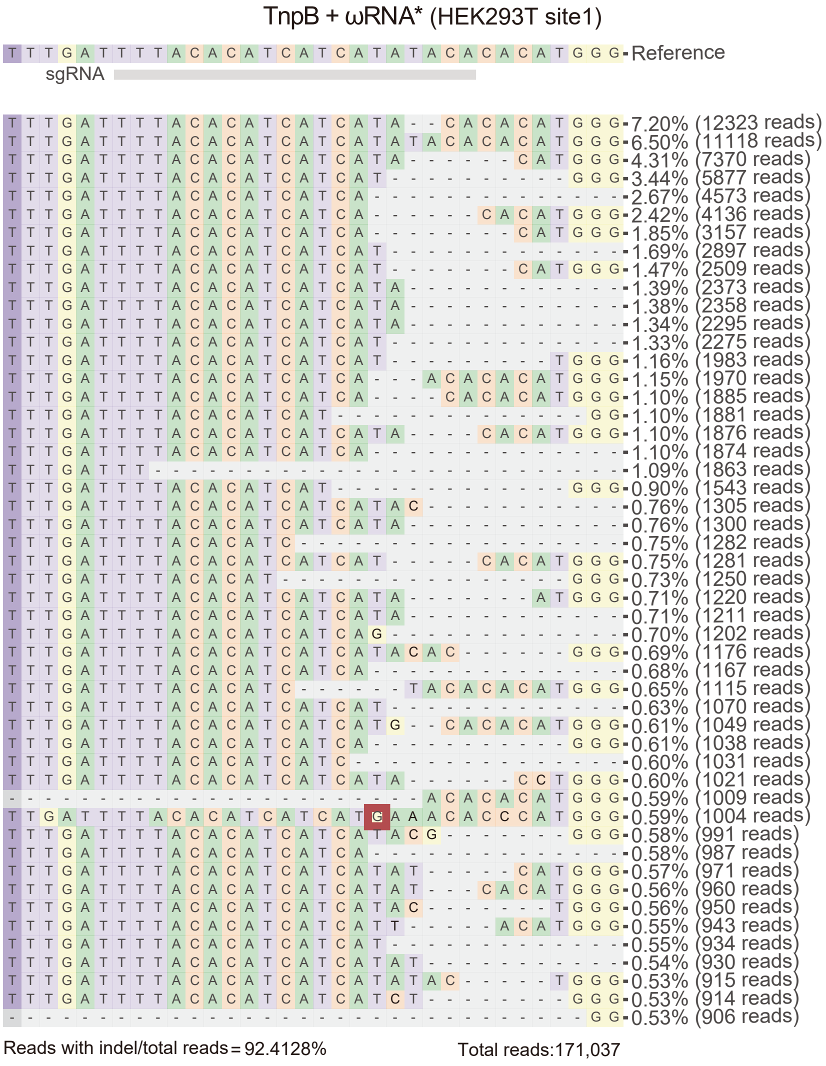
TnpB + ωRNA* (HEK293T site1)
TTTGATTTTACACATCATCATATACACACATGGG Reference
sgRNA
7.20% (12323 reads)
6.50% (11118 reads)
4.31% (7370 reads)
3.44% (5877 reads)
2.67% (4573 reads)
2.42% (4136 reads)
1.85% (3157 reads)
1.69% (2897 reads)
1.47% (2509 reads)
1.39% (2373 reads)
1.38% (2358 reads)
1.34% (2295 reads)
1.33% (2275 reads)
1.16% (1983 reads)
1.15% (1970 reads)
1.10% (1885 reads)
1.10% (1881 reads)
1.10% (1876 reads)
1.10% (1874 reads)
1.09% (1863 reads)
0.90% (1543 reads)
0.76% (1305 reads)
0.76% (1300 reads)
0.75% (1282 reads)
0.75% (1281 reads)
0.73% (1250 reads)
0.71% (1220 reads)
0.71% (1211 reads)
0.70% (1202 reads)
0.69% (1176 reads)
0.68% (1167 reads)
0.65% (1115 reads)
0.63% (1070 reads)
0.61% (1049 reads)
0.61% (1038 reads)
0.60% (1031 reads)
0.60% (1021 reads)
0.59% (1009 reads)
0.59% (1004 reads)
0.58% (991 reads)
0.58% (987 reads)
0.57% (971 reads)
0.56% (960 reads)
0.56% (950 reads)
0.55% (943 reads)
0.55% (934 reads)
0.54% (930 reads)
0.53% (915 reads)
0.53% (914 reads)
0.53% (906 reads)
Reads with indel/total reads = 92.4128%
Total reads:171,037

Fig.S7

Fig. S7. Deep-seq reads analysis results for site1 edited with TnpB-ωRNA/ωRNA* in HEK293T cells.

a. Deep-seq reads analysis results for HEK1-site1 by TnpB-ωRNA. **b.** Deep-seq reads analysis results for HEK1-site1 by TnpB-ωRNA*.

a

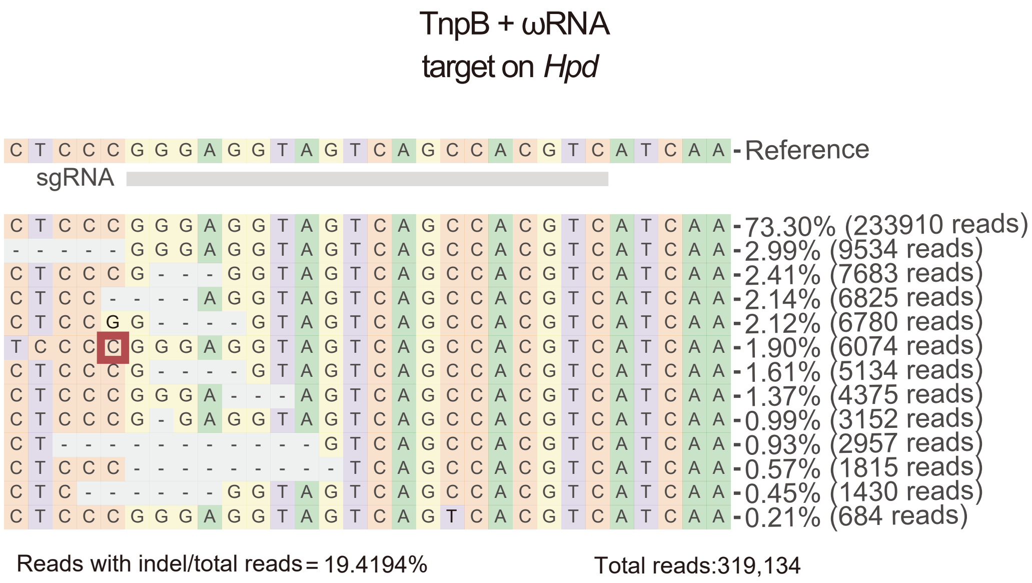
TnpB + ωRNA
target on *Hpd*
C T C C C G G G A G G T A G T C A G C C A C G T C A T C A A - Reference
sgRNA
-73.30% (233910 reads)
-2.99% (9534 reads)
-2.41% (7683 reads)
-2.14% (6825 reads)
-2.12% (6780 reads)
-1.90% (6074 reads)
-1.61% (5134 reads)
-1.37% (4375 reads)
-0.99% (3152 reads)
-0.93% (2957 reads)
-0.57% (1815 reads)
-0.45% (1430 reads)
-0.21% (684 reads)
Reads with indel/total reads = 19.4194%
Total reads:319,134

b

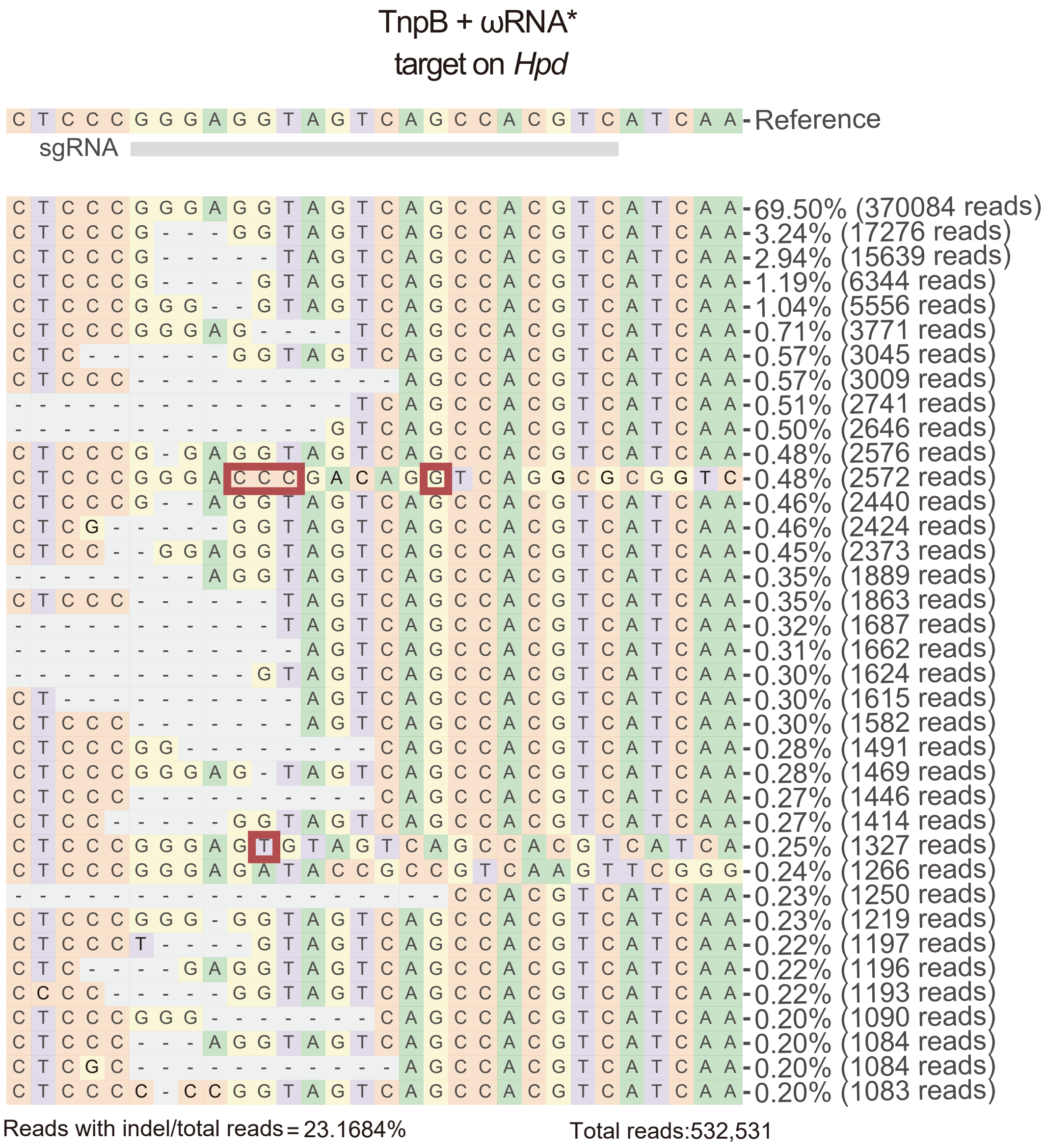
TnpB + ωRNA*
target on *Hpd*
C T C C C G G G A G G T A G T C A G C C A C G T C A T C A A - Reference
sgRNA
-69.50% (370084 reads)
-3.24% (17276 reads)
-2.94% (15639 reads)
-1.19% (6344 reads)
-1.04% (5556 reads)
-0.71% (3771 reads)
-0.57% (3045 reads)
-0.57% (3009 reads)
-0.51% (2741 reads)
-0.50% (2646 reads)
-0.48% (2576 reads)
-0.48% (2572 reads)
-0.46% (2440 reads)
-0.46% (2424 reads)
-0.45% (2373 reads)
-0.35% (1889 reads)
-0.35% (1863 reads)
-0.32% (1687 reads)
-0.31% (1662 reads)
-0.30% (1624 reads)
-0.30% (1615 reads)
-0.30% (1582 reads)
-0.28% (1491 reads)
-0.28% (1469 reads)
-0.27% (1446 reads)
-0.27% (1414 reads)
-0.25% (1327 reads)
-0.24% (1266 reads)
-0.23% (1250 reads)
-0.23% (1219 reads)
-0.22% (1197 reads)
-0.22% (1196 reads)
-0.22% (1193 reads)
-0.20% (1090 reads)
-0.20% (1084 reads)
-0.20% (1084 reads)
-0.20% (1083 reads)
Reads with indel/total reads = 23.1684%
Total reads:532,531

Fig.S8

Fig. S8. Deep-seq reads analysis results for Hpd edited with TnpB-ωRNA/ωRNA* in N2a cells.

a. Deep-seq reads analysis results for *Hpd* by TnpB-ωRNA. **b.** Deep-seq reads analysis results for *Hpd* by TnpB-ωRNA*.

a

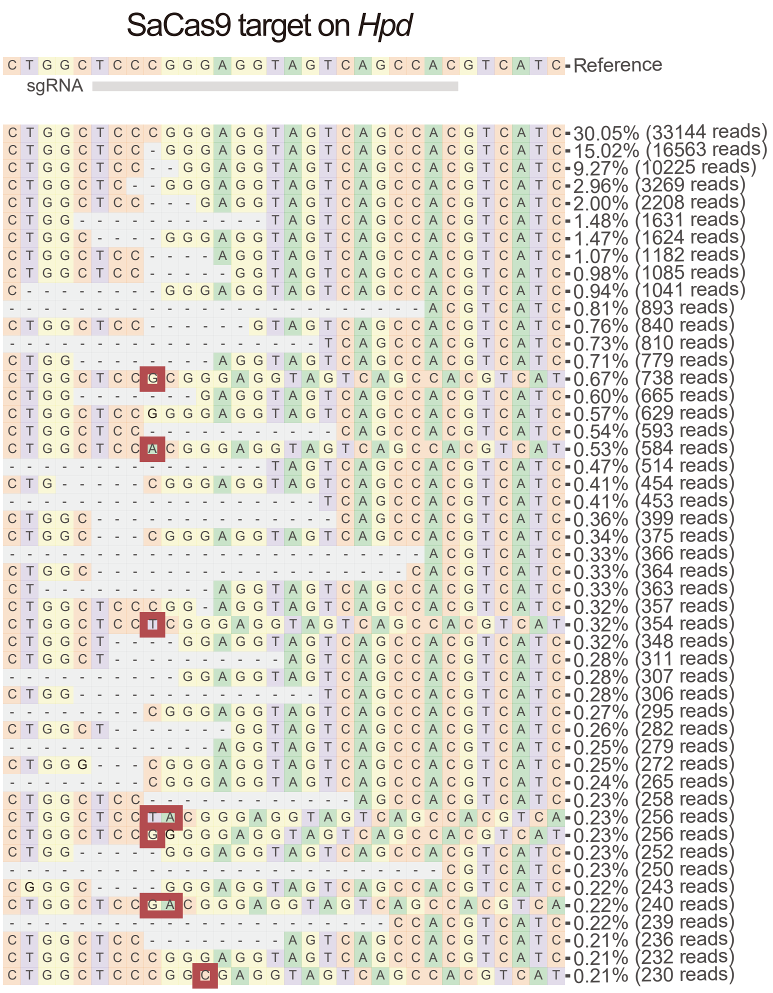

Reads with indel/total reads=65.8201% Total reads:110,290

b

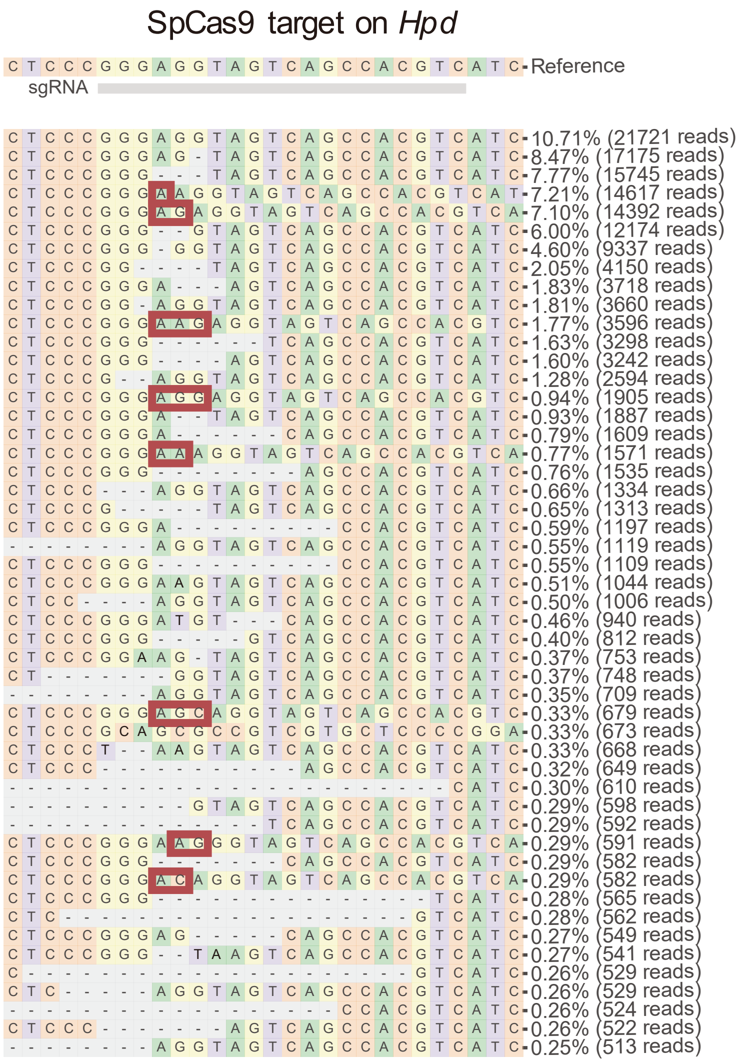

Reads with indel/total reads = 87.0082% Total reads:202,807

Fig.S9

Fig. S9. Deep-seq reads analysis results for Hpd edited with SaCas9 and SpCas9 in N2a cells.

a. Deep-seq reads analysis results for *Hpd* by SaCas9. **b.** Deep-seq reads analysis results for *Hpd* by SpCas9.

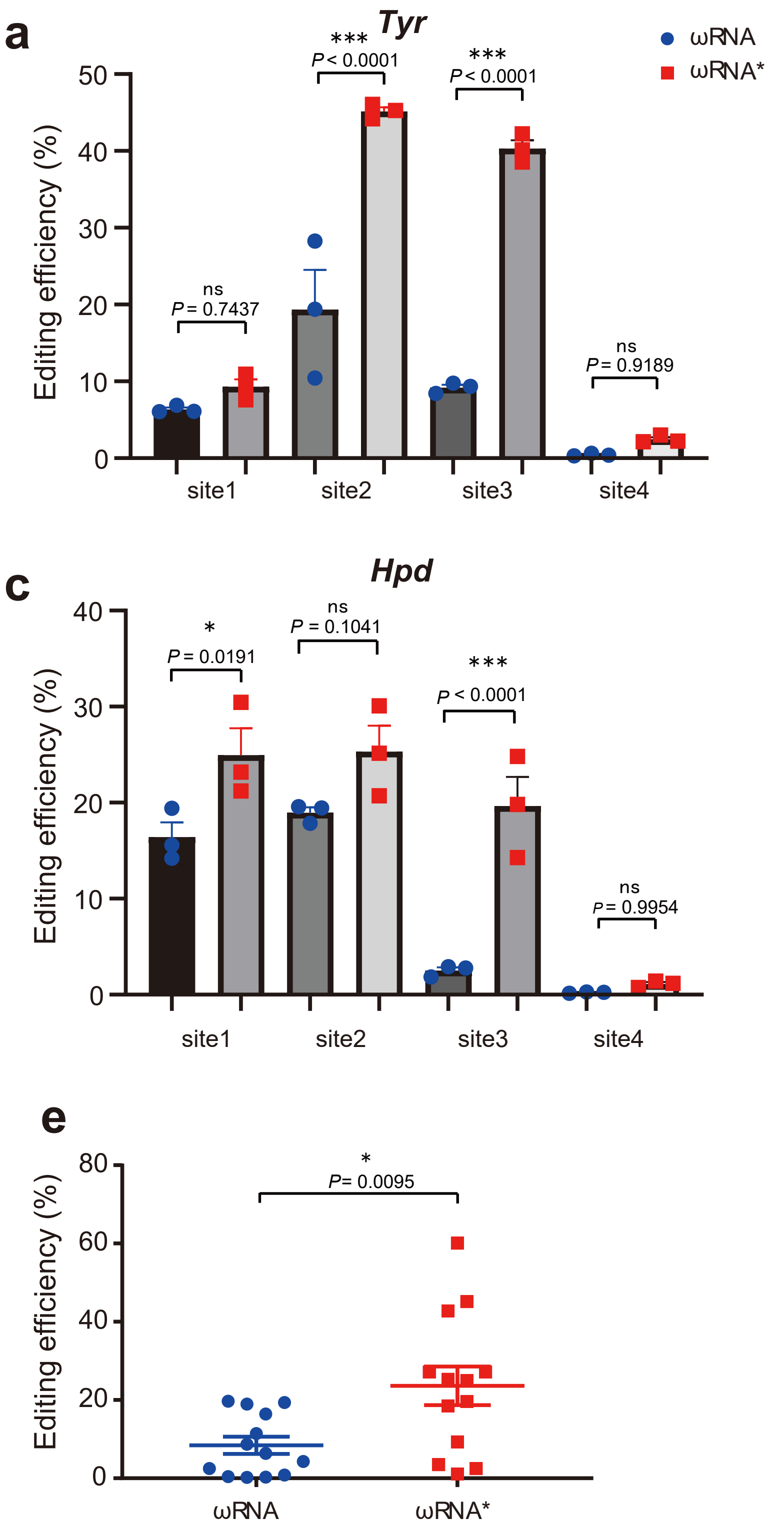
a
Tyr
ωRNA
ωRNA*
Editing efficiency (%)
ns P = 0.7437
*** P < 0.0001
*** P < 0.0001
ns P = 0.9189
site1
site2
site3
site4
b
Klkb1
Editing efficiency (%)
*** P< 0.0001
*** P< 0.0001
*** P< 0.0001
Site1
Site2
Site3
c
Hpd
Editing efficiency (%)
* P = 0.0191
ns P = 0.1041
*** P < 0.0001
ns P = 0.9954
site1
site2
site3
site4
d
Pcsk9
Editing efficiency (%)
ns P= 0.9954
*** P< 0.0001
* P< 0.0006
site2
site3
site4
e
Editing efficiency (%)
* P= 0.0095
ωRNA
ωRNA*

Fig.S10

Fig. S10. Characterization of gene editing activity for engineered TnpB-ωRNA system in mouse N2a cells.

a-d. Efficiency comparison using cognate and engineered ωRNA for mouse *Klkb1, Tyr, Hpd,* and *Pcsk9* gene editing. **b.** Summary statistic results for gene editing activity characterization of cognate and engineered ωRNA in N2a. Data are represented as means ± SEM. A dot represents a biological replicate. Significant differences between conditions are indicated by asterisk. Data are represented as means ± SEM. A dot represents a biological replicate (n=3 or more). Unpaired two-tailed Student's t tests. (* $P < 0.05$, ** $P<0.01$, *** $P < 0.001$, **** $P < 0.0001$, NS non-significant.). Source data are provided in the Source Data File.

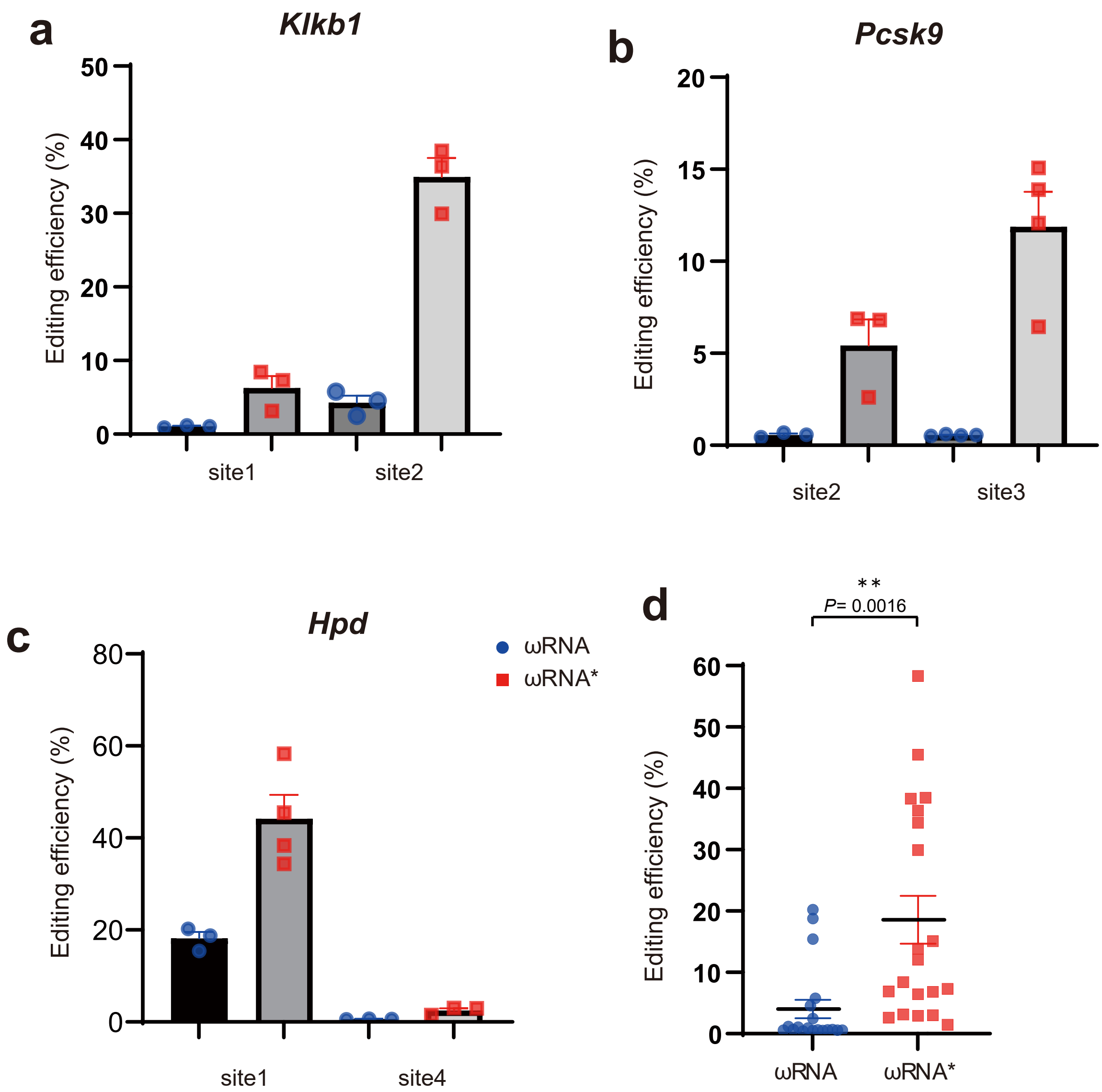
a
Klkb1
Editing efficiency (%)
50
40
30
20
10
0
site1
site2
b
Pcsk9
Editing efficiency (%)
20
15
10
5
0
site2
site3
c
Hpd
ωRNA
ωRNA*
Editing efficiency (%)
80
60
40
20
0
site1
site4
d
**
P= 0.0016
Editing efficiency (%)
60
50
40
30
20
10
0
ωRNA
ωRNA*

Fig.S11

Fig. S11. In vivo characterization of gene editing activity for the engineered TnpB-ωRNA* system delivered by AAV in mice.

a-c. In vivo gene editing efficiency comparison for mouse *Klkb1, Tyr, Hpd,* and *Pcsk9* genes using cognate and engineered ωRNA. **d.** Summary statistic results for gene editing activity characterization of cognate and engineered ωRNA via in vivo delivery with AAV. Data are represented as means ± SEM. A dot represents a biological replicate. Significant differences between conditions are indicated by asterisk. Data are represented as means ± SEM. A dot represents a biological replicate (n=3 or more). Unpaired two-tailed Student's t tests. (* $P < 0.05$, ** $P<0.01$, *** $P < 0.001$, **** $P < 0.0001$, NS non-significant.). Source data are provided in the Source Data File.

a b

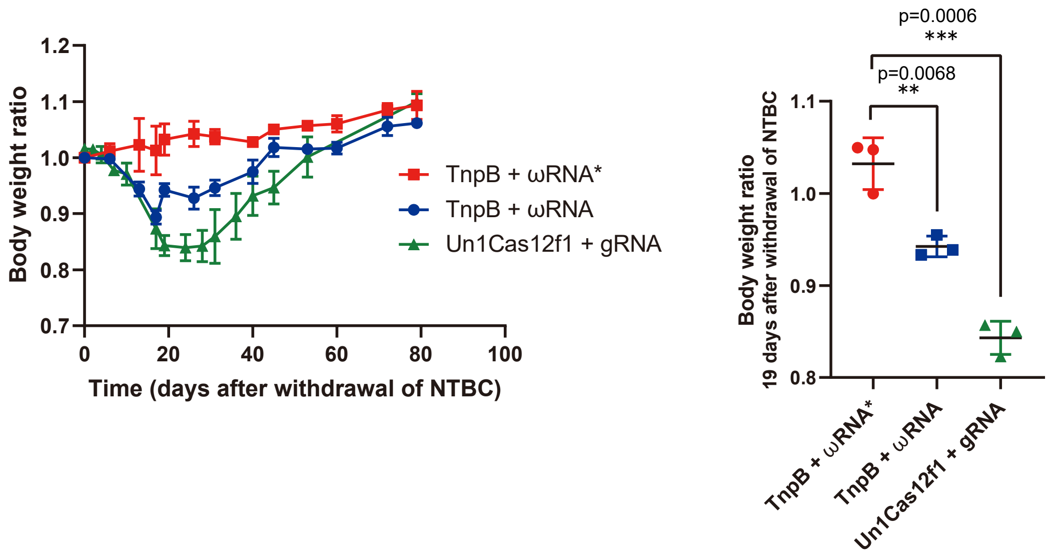

Body weight ratio
1.2
1.1
1.0
0.9
0.8
0.7
0
20
40
60
80
100
Time (days after withdrawal of NTBC)
TnpB + ωRNA*
TnpB + ωRNA
Un1Cas12f1 + gRNA
p=0.0006
***
p=0.0068
**
Body weight ratio
19 days after withdrawal of NTBC
TnpB + ωRNA*
TnpB + ωRNA
Un1Cas12f1 + gRNA

Fig.S12

Fig. S12. Comparison of therapeutic efficacy between TnpB and Un1Cas12f1 in HT1 mice.

a. Body weight change for HT1 mice in different treatment groups. **b.** Body weight ratio for Un1Cas12f1 or TnpB-ωRNA versus TnpB-ωRNA* treated mice in 19-day after NTBC withdrawal. Data are represented as means ± SEM. A dot represents a biological replicate (n=3 or more). Unpaired two-tailed Student's t tests. (* $P < 0.05$, ** $P<0.01$, *** $P < 0.001$, **** $P < 0.0001$, NS non-significant.). Source data are provided in the Source Data File.

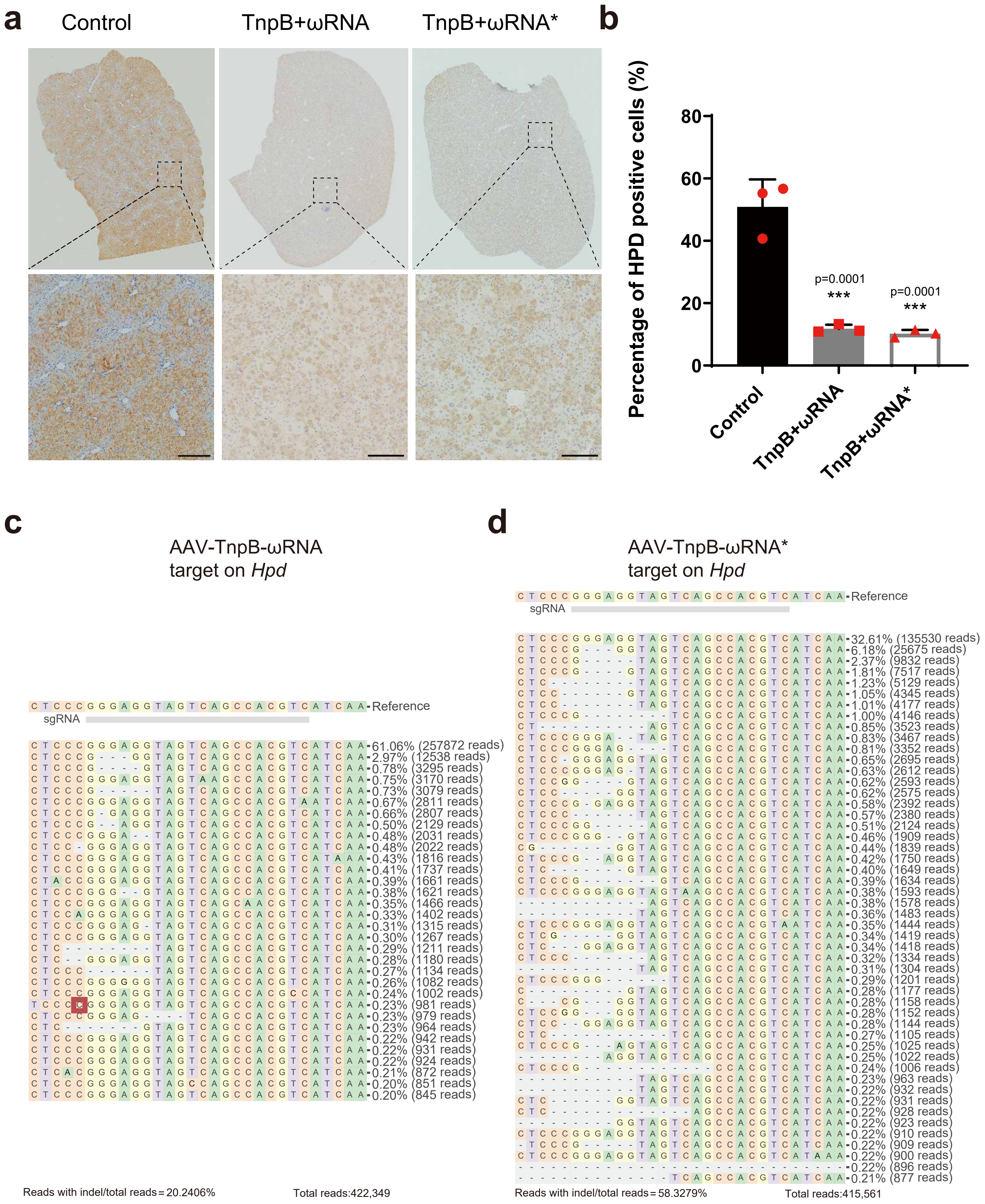
a
Control
TnpB+ωRNA
TnpB+ωRNA*
b
Percentage of HPD positive cells (%)
80
60
40
20
0
p=0.0001
***
p=0.0001
***
Control
TnpB+ωRNA
TnpB+ωRNA*
c
AAV-TnpB-ωRNA
target on Hpd
C T C C C G G G A G G T A G T C A G C C A C G T C A T C A A
Reference
sgRNA
61.06% (257872 reads)
2.97% (12538 reads)
0.78% (3295 reads)
0.75% (3170 reads)
0.73% (3079 reads)
0.67% (2811 reads)
0.66% (2807 reads)
0.50% (2129 reads)
0.48% (2031 reads)
0.48% (2022 reads)
0.43% (1816 reads)
0.41% (1737 reads)
0.39% (1661 reads)
0.38% (1621 reads)
0.35% (1466 reads)
0.33% (1402 reads)
0.31% (1315 reads)
0.30% (1267 reads)
0.29% (1211 reads)
0.28% (1180 reads)
0.27% (1134 reads)
0.26% (1082 reads)
0.24% (1002 reads)
0.23% (981 reads)
0.23% (979 reads)
0.23% (964 reads)
0.22% (942 reads)
0.22% (931 reads)
0.22% (924 reads)
0.21% (872 reads)
0.20% (851 reads)
0.20% (845 reads)
Reads with indel/total reads = 20.2406%
Total reads:422,349
d
AAV-TnpB-ωRNA*
target on Hpd
C T C C C G G G A G G T A G T C A G C C A C G T C A T C A A
Reference
sgRNA
32.61% (135530 reads)
6.18% (25675 reads)
2.37% (9832 reads)
1.81% (7517 reads)
1.23% (5129 reads)
1.05% (4345 reads)
1.01% (4177 reads)
1.00% (4146 reads)
0.85% (3523 reads)
0.83% (3467 reads)
0.81% (3352 reads)
0.65% (2695 reads)
0.63% (2612 reads)
0.62% (2593 reads)
0.62% (2575 reads)
0.58% (2392 reads)
0.57% (2380 reads)
0.51% (2124 reads)
0.46% (1909 reads)
0.44% (1839 reads)
0.42% (1750 reads)
0.40% (1649 reads)
0.39% (1634 reads)
0.38% (1593 reads)
0.38% (1578 reads)
0.36% (1483 reads)
0.35% (1444 reads)
0.34% (1419 reads)
0.34% (1418 reads)
0.32% (1334 reads)
0.31% (1304 reads)
0.29% (1201 reads)
0.28% (1177 reads)
0.28% (1158 reads)
0.28% (1152 reads)
0.28% (1144 reads)
0.27% (1105 reads)
0.25% (1025 reads)
0.25% (1022 reads)
0.24% (1006 reads)
0.23% (963 reads)
0.22% (932 reads)
0.22% (931 reads)
0.22% (928 reads)
0.22% (923 reads)
0.22% (910 reads)
0.22% (909 reads)
0.22% (900 reads)
0.22% (896 reads)
0.21% (877 reads)
Reads with indel/total reads = 58.3279%
Total reads:415,561

Fig.S13

Fig. S13. Immunostaining and gene editing analysis for HPD in AAV-TnpB-ωRNA treated mouse liver.

a. Hpd immunostaining analysis in $Fah^{-/-}$ mice treated with or without AAV-TnpB-ωRNA. **b.** Deep-seq results for *Hpd* gene editing by AAV-TnpB. Data are represented as means ± SEM. **c.** Deep-seq reads analysis results for *Hpd* by AAV-TnpB-ωRNA. **d.** Deep-seq reads analysis results for *Hpd* by AAV-TnpB-ωRNA*. A dot represents a biological replicate (n=3 or more). Unpaired two-tailed Student's t tests. (* P < 0.05, ** P<0.01, *** P < 0.001, **** P < 0.0001, NS non-significant.). Source data are provided in the Source Data File.

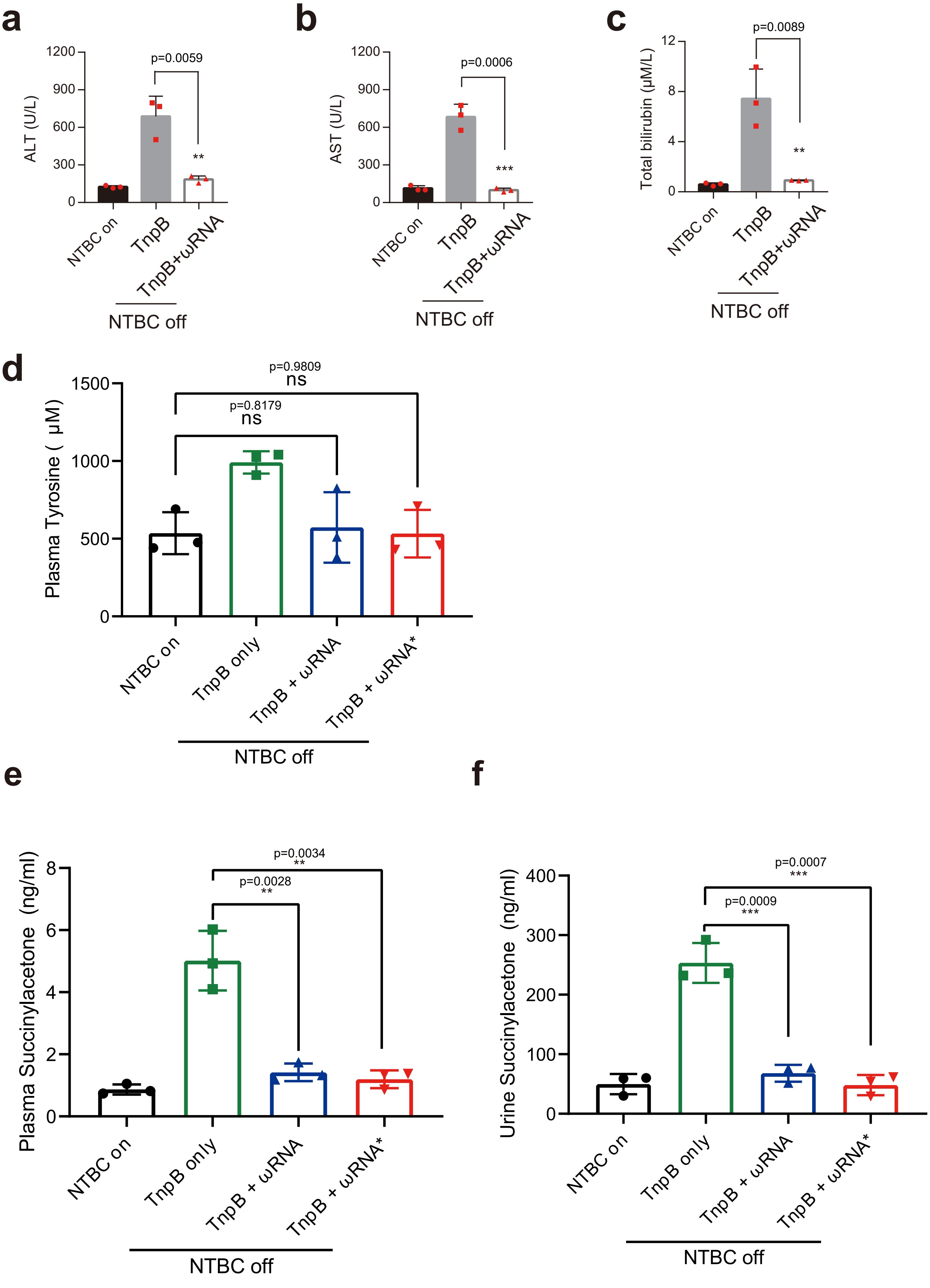

a
ALT (U/L)
1200
900
600
300
0
p=0.0059
**
NTBC on
TnpB
TnpB+ωRNA
NTBC off
b
AST (U/L)
1200
900
600
300
0
p=0.0006
***
NTBC on
TnpB
TnpB+ωRNA
NTBC off
c
Total bilirubin (μM/L)
12
8
4
0
p=0.0089
**
NTBC on
TnpB
TnpB+ωRNA
NTBC off
d
Plasma Tyrosine (μM)
1500
1000
500
0
p=0.9809
ns
p=0.8179
ns
NTBC on
TnpB only
TnpB + ωRNA
TnpB + ωRNA*
NTBC off
e
Plasma Succinylacetone (ng/ml)
8
6
4
2
0
p=0.0034
**
p=0.0028
**
NTBC on
TnpB only
TnpB + ωRNA
TnpB + ωRNA*
NTBC off
f
Urine Succinylacetone (ng/ml)
400
300
200
100
0
p=0.0007
***
p=0.0009
***
NTBC on
TnpB only
TnpB + ωRNA
TnpB + ωRNA*
NTBC off

Fig.S14

Fig. S14. Plasma and urine biochemical analysis for AAV-TnpB-ωRNA treated mice.

a-d. Biochemical analysis of serum indicators for liver metabolic function in TnpB-treated or untreated mice (n=3). Liver damage markers alanine aminotransferase (ALT), aspartate aminotransferase (AST), total bilirubin and tyrosine were measured in peripheral blood from $Fah^{-/-}$ mice injected with AAV-TnpB without or with ωRNA (NTBC off, day 30). **e,f.** Plasma and urine succinylacetone analysis for untreated and treated HT1 mice. $Fah^{-/-}$ mice on NTBC water (NTBC on) served as a control. Data are represented as means ± SEM. A dot represents a biological replicate. Significant differences between conditions are indicated by asterisk. Data are represented as means ± SEM. A dot represents a biological replicate (n=3 or more). Unpaired two-tailed Student's t tests. (* P < 0.05, ** P<0.01, *** P < 0.001, **** P < 0.0001, NS non-significant.). Source data are provided in the Source Data File.

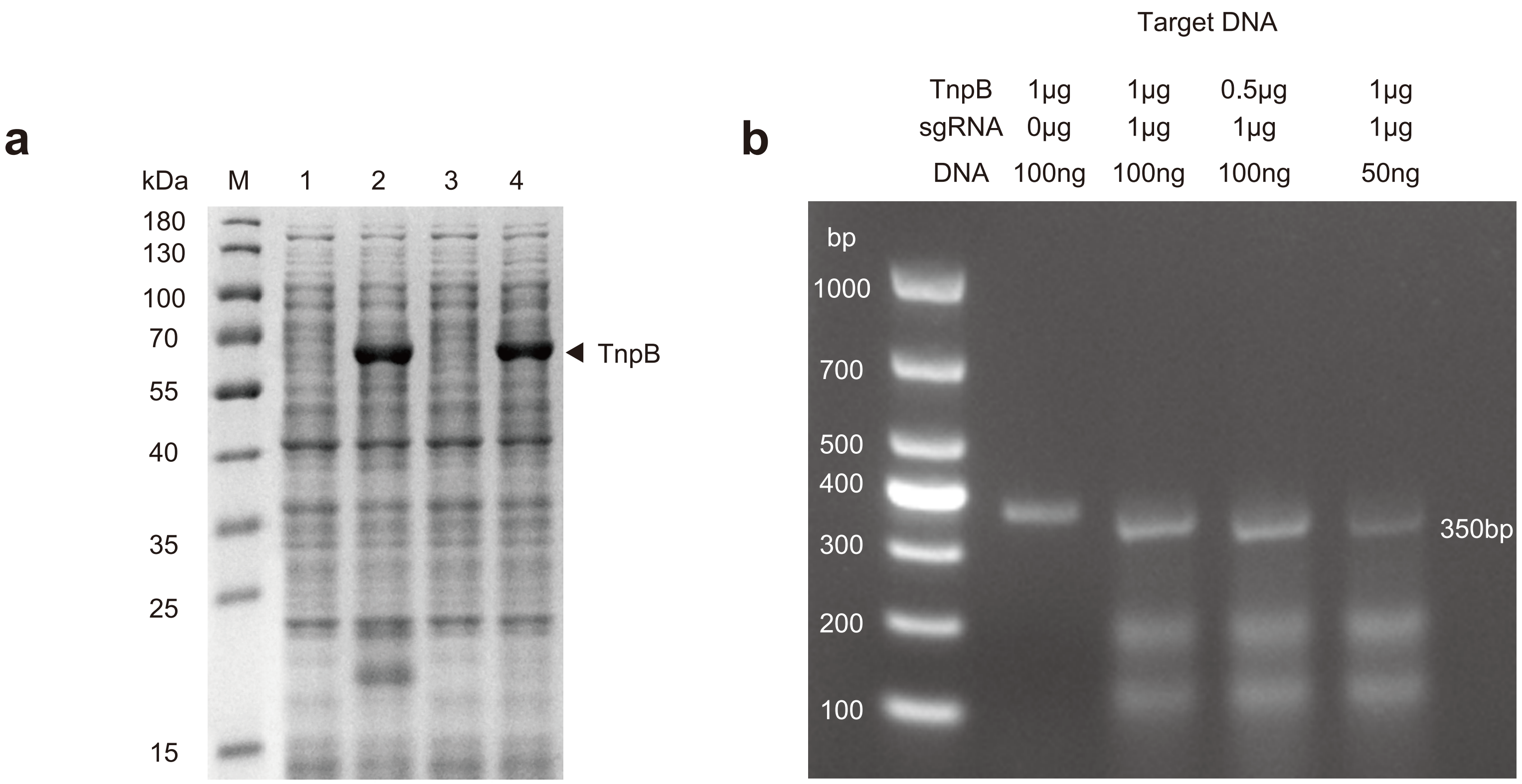
a
kDa
M
1
2
3
4
180
130
100
70
55
40
35
25
15
TnpB
b
Target DNA
TnpB
1μg
1μg
0.5μg
1μg
sgRNA
0μg
1μg
1μg
1μg
DNA
100ng
100ng
100ng
50ng
bp
1000
700
500
400
300
200
100
350bp

Fig.S15

Fig. S15. TnpB expression in E. coli. and in vitro DNA cleavage analysis. **a.** TnpB expression analysis by SDS-PAGE for E. coli. with or without IPTG induction. **b.** In vitro biochemical analysis of endonuclease activity of purified TnpB protein with ωRNA.

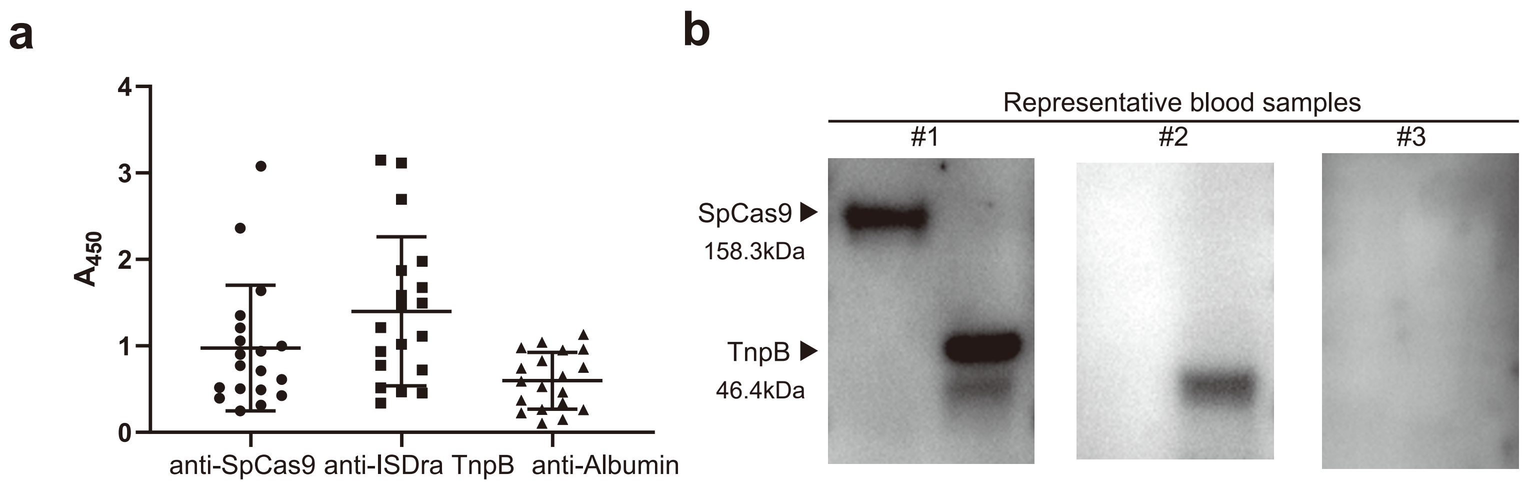
a
4
3
2
1
0
A450
anti-SpCas9
anti-ISDra TnpB
anti-Albumin
b
Representative blood samples
#1
#2
#3
SpCas9
158.3kDa
TnpB
46.4kDa

Fig.S16

Fig. S16. Antibody analysis in the human blood for TnpB and SpCas9. **a.** ELISA assay for anti-TnpB and -SpCas9 activity in human blood. **b.** Western blot for anti-TnpB and -SpCas9 activity in representative blood samples. Source data are provided in the Source Data File.

Figure exemplifying the gating strategy for Flow Cytometry

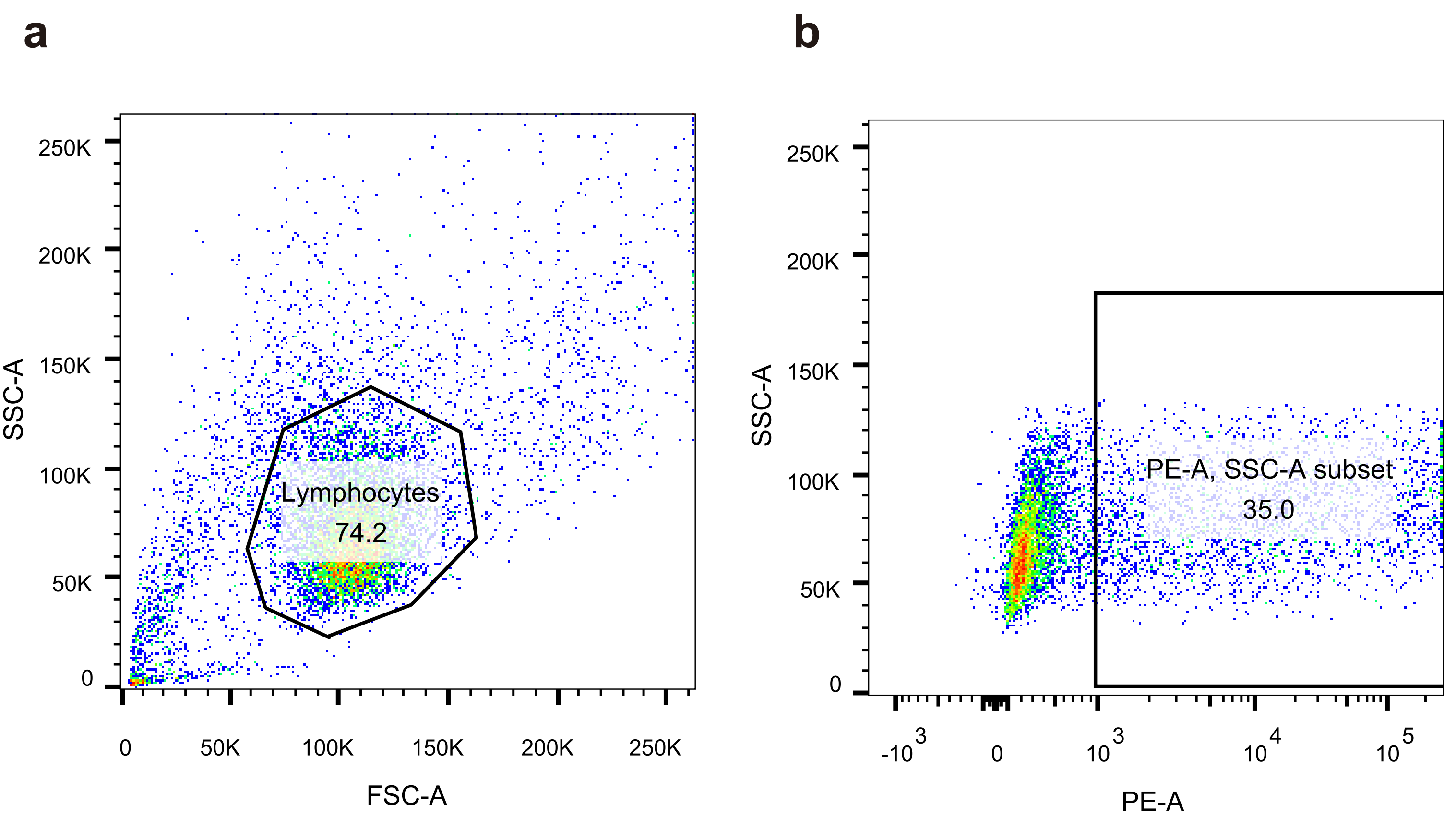

Fig.S17

Fig. S17. Figure exemplifying the gating strategy for Flow Cytometry.
a. Single cell gating strategy for cell population. **b.** Fluorescent cells ($mCherry^{+}$) gating strategy.

Supplementary Table S1. Gene editing efficiency for TnpB targeting human 14 sites.

Target site	TnpB-ωRNA			TnpB-ωRNA*		
Site1	80.9538	79.7051	77.9616	92.4128	92.7251	88.9477
Site2	91.1666	84.4706	86.6302	87.5109	91.692	87.0071
Site3	57.344	58.412	57.7154	83.7087	89.3263	78.8584
Site4	38.84	37.6139	42.7442	69.6037	72.8125	70.9723
Site5	12.5092	13.0002	19.1259	26.4571	31.0378	33.6545
Site6	54.4544	37.7141	50.0122	88.7723	90.357	88.4455
Site7	93.4096	96.0689	89.3796	91.7691	96.3689	95.9455
Site8	88.778	85.8337	86.537	91.9339	92.7217	88.5393
Site9	81.6767	91.7921	86.5068	86.3587	80.7439	81.0622
Site10	44.6191	52.511	51.8882	63.8667	64.4691	69.6262
Site11	40.9502	32.3023	36.2421	47.3455	42.452	49.8979
Site12	52.1795	51.2256	51.1079	41.4787	45.473	34.213
Site13	54.378	42.4125	53.2031	82.4258	81.97	78.2429
Site14	53.6541	61.4214	56.5062	83.8742	79.3327	81.1779

Supplementary Table S2. Target sgRNA and primer sequence.

sgRNA	Target site sequences (5’-3’)	Primer sequence
HEK1-site1	GTCTTATCACCCTATAGAGT	TCAA GTCTTATCACCCTATAGAGT
		GGCC ACTCTATAGGGTGATAAGAC
HEK1-site2	AATGGAGGTCTGGTGAGTAT	TCAA AATGGAGGTCTGGTGAGTAT
		GGCC ATACTCACCAGACCTCCATT
HEK1-site3	TGTTTTATTTATTACACACA	TCAA TGTTTTATTTATTACACACA
		GGCC TGTGTGTAATAAATAAAACA
HEK1-site4	TTTACACATCATCATATACA	TCAA TTTACACATCATCATATACA
		GGCC TGTATATGATGATGTGTAAA
HEK1-site5	CTTTTCAAAATTACTTTGCA	TCAA CTTTTCAAAATTACTTTGCA
		GGCC TGCAAAGTAATTTTGAAAAG
HEK1-site6	AGAAGTGAGATGGCTCCAAA	TCAA AGAAGTGAGATGGCTCCAAA
		GGCC TTTGGAGCCATCTCACTTCT
HEK1-site7	GACCCAAAGAAATGTATTCC	TCAA GACCCAAAGAAATGTATTCC
		GGCC GGAATACATTTCTTTGGGTC
HEK1-site8	ATTCAAAAACACGCAAACCC	TCAA ATTCAAAAACACGCAAACCC
		GGCC GGGTTTGCGTGTTTTTGAAT
HEK1-site9	GTGATTTCTTGGAAATAGTT	TCAA GTGATTTCTTGGAAATAGTT
		GGCC AACTATTTCCAAGAAATCAC
HEK1-site10	AAAGAAGTCTACTTTGACTT	TCAA AAAGAAGTCTACTTTGACTT
		GGCC AAGTCAAAGTAGACTTCTTT
HEK1-site11	TCCTTTGCCAGGTTTCTGCA	TCAA TCCTTTGCCAGGTTTCTGCA

		GGCC TGCAGAAACCTGGCAAAGGA
HEK1-site12	TCTGCACAGAGCTCCAAATG	TCAA TCTGCACAGAGCTCCAAATG
		GGCC CATTTGGAGCTCTGTGCAGA
HEK1-site13	AACTAGACCTGTTGCTCAAA	TCAA AACTAGACCTGTTGCTCAAA
		GGCC TTTGAGCAACAGGTCTAGTT
HEK1-site14	TAATTAGAGCATAAATAAGA	TCAA TAATTAGAGCATAAATAAGA
		GGCC TCTTATTTATGCTCTAATTA
HEK1-site15	TTGGGGCTAGCTGTATTCTC	TCAA TTGGGGCTAGCTGTATTCTC
		GGCC GAGAATACAGCTAGCCCCAA
HEK1-site16	GGGTTATGAATATTGACACA	TCAA GGGTTATGAATATTGACACA
		GGCC TGTGTCAATATTCATAACCC
HEK1-site17	TCAAGTACCACCAGTTTTAT	TCAA TCAAGTACCACCAGTTTTAT
		GGCC ATAAAACTGGTGGTACTTGA
HEK1-site18	AACTTTAGATGTCTGTTTCC	TCAA AACTTTAGATGTCTGTTTCC
		GGCC GGAAACAGACATCTAAAGTT
HEK1-site19	AGAACACCCATAAGAACAAC	TCAA AGAACACCCATAAGAACAAC
		GGCC GTTGTTCTTATGGGTGTTCT
HEK1-site20	GAAAAGATTACAGAATCAGG	TCAA GAAAAGATTACAGAATCAGG
		GGCC CCTGATTCTGTAATCTTTTC
HEK1-site21	TATGTCTAGGTGAACTGGTA	TCAA TATGTCTAGGTGAACTGGTA
		GGCC TACCAGTTCACCTAGACATA
HEK1-site22	AGATGATGTTTCCACACATA	TCAA AGATGATGTTTCCACACATA
		GGCC TATGTGTGGAAACATCATCT
HEK1-site23	TTTTAAGGAATTTGACATAT	TCAA TTTTAAGGAATTTGACATAT
		GGCC ATATGTCAAATTCCTTAAAA
HEK1-site24	CCAAGTTCAAAAGTTGTAAT	TCAA CCAAGTTCAAAAGTTGTAAT
		GGCC ATTACAACTTTTGAACTTGG
HEK1-site25	CTGCCTTCAGAAAGCACCTT	TCAA CTGCCTTCAGAAAGCACCTT
		GGCC AAGGTGCTTTCTGAAGGCAG
HEK1-site26	AAAAATGCATGAAGCTCCTT	TCAA AAAAATGCATGAAGCTCCTT
		GGCC AAGGAGCTTCATGCATTTTT
HEK1-site27	TAAGGAACTAGAATCTAAAA	TCAA TAAGGAACTAGAATCTAAAA
		GGCC TTTTAGATTCTAGTTCCTTA
HEK1-site28	CCTTACTGACTTTCAGCTTT	TCAA CCTTACTGACTTTCAGCTTT
		GGCC AAAGCTGAAAGTCAGTAAGG
HEK1-site29	GAGTCCAGTCAGAAAGCAGA	TCAA GAGTCCAGTCAGAAAGCAGA
		GGCC TCTGCTTTCTGACTGGACTC
HEK1-site30	TTCAAGAATATTTTAGCACA	TCAA TTCAAGAATATTTTAGCACA
		GGCC TGTGCTAAAATATTCTTGAA
HEK1-site31	TCTCTCATAATATTTCATTT	TCAA TCTCTCATAATATTTCATTT

		GGCC AAATGAAATATTATGAGAGA
HEK2-site1	ATAAACATTTTATTAACATG	TCAA ATAAACATTTTATTAACATG
		GGCC CATGTTAATAAAATGTTTAT
HEK2-site2	TGAGATTAATTCACATGTGA	TCAA TGAGATTAATTCACATGTGA
		GGCC TCACATGTGAATTAATCTCA
HEK2-site3	TCATGTGTTCAAACAGTTTC	TCAA TCATGTGTTCAAACAGTTTC
		GGCC GAAACTGTTTGAACACATGA
HEK2-site4	GTTGTAGGGTTTTTTGTTTG	TCAA GTTGTAGGGTTTTTTGTTTG
		GGCC CAAACAAAAAACCCTACAAC
HEK2-site5	TCAAAATGATCCTTTATGTA	TCAA TCAAAATGATCCTTTATGTA
		GGCC TACATAAAGGATCATTTTGA
HEK2-site6	TGCAACAAACCAACCATTTT	TCAA TGCAACAAACCAACCATTTT
		GGCC AAAATGGTTGGTTTGTTGCA
HEK2-site7	CCTCAGGGTGGTCAGGGCCA	TCAA CCTCAGGGTGGTCAGGGCCA
		GGCC TGGCCCTGACCACCCTGAGG
HEK2-site8	AAATATGTAATTAAATGTCT	TCAA AAATATGTAATTAAATGTCT
		GGCC AGACATTTAATTACATATTT
HEK2-site9	GAATCAGTGCTGGAGAATGG	TCAA GAATCAGTGCTGGAGAATGG
		GGCC CCATTCTCCAGCACTGATTC
HEK2-site10	GCTTTTTTTCTGCTTCTCCA	TCAA GCTTTTTTTCTGCTTCTCCA
		GGCC TGGAGAAGCAGAAAAAAAGC
HEK2-site11	CTCTGATTTTCATGCAGGTG	TCAA CTCTGATTTTCATGCAGGTG
		GGCC CACCTGCATGAAAATCAGAG
HEK2-site12	CCGTAGCCAGGAAGTTAGAT	TCAA CCGTAGCCAGGAAGTTAGAT
		GGCC ATCTAACTTCCTGGCTACGG
HEK2-site13	TAATGGAGACATTGCCATGC	TCAA TAATGGAGACATTGCCATGC
		GGCC GCATGGCAATGTCTCCATTA
HEK2-site14	CACCTGCCCAAATGTGAGGA	TCAA CACCTGCCCAAATGTGAGGA
		GGCC TCCTCACATTTGGGCAGGTG
HEK2-site15	TTCGCCAGCTCTGATGAGGC	TCAA TTCGCCAGCTCTGATGAGGC
		GGCC GCCTCATCAGAGCTGGCGAA
HEK2-site16	ATGGGGATAGGTGGAGACTA	TCAA ATGGGGATAGGTGGAGACTA
		GGCC TAGTCTCCACCTATCCCCAT
HEK3-site1	TTATTAGCATAAGAGTGTCT	TCAA TTATTAGCATAAGAGTGTCT
		GGCC AGACACTCTTATGCTAATAA
HEK3-site2	CTTTAGTAAAGACAGGCAAC	TCAA CTTTAGTAAAGACAGGCAAC
		GGCC GTTGCCTGTCTTTACTAAAG
HEK3-site3	TACACAGCTGACTCACTCTG	TCAA TACACAGCTGACTCACTCTG
		GGCC

		CAGAGTGAGTCAGCTGTGTA
HEK3-site4	CAATGCACTTTTAAAATTGT	TCAA CAATGCACTTTTAAAATTGT
		GGCC ACAATTTTAAAAGTGCATTG
HEK3-site5	CAAATTCATCATCTCACCTA	TCAA CAAATTCATCATCTCACCTA
		GGCC TAGGTGAGATGATGAATTTG
HEK3-site6	GCATCCCCCATCCACTATAA	TCAA GCATCCCCCATCCACTATAA
		GGCC TTATAGTGGATGGGGGATGC
HEK3-site7	GGCTCAGCTCAGGAGCACCC	TCAA GGCTCAGCTCAGGAGCACCC
		GGCC GGGTGCTCCTGAGCTGAGCC
HEK3-site8	TTTTCCAGAGTTGAGATGAT	TCAA TTTTCCAGAGTTGAGATGAT
		GGCC ATCATCTCAACTCTGGAAAA
HEK3-site9	CTTCCTCAGATGTTTAGGCA	TCAA CTTCCTCAGATGTTTAGGCA
		GGCC TGCCTAAACATCTGAGGAAG
HEK3-site10	GTCTGTACTTAATAACGAAT	TCAA GTCTGTACTTAATAACGAAT
		GGCC ATTCGTTATTAAGTACAGAC
hVEGFA-site1	GAGGGGAGCTGTGGCTCAGA	TCAA GAGGGGAGCTGTGGCTCAGA
		GGCC TCTGAGCCACAGCTCCCCTC
hVEGFA-site2	GTCTGCAGGCCAGATGAGGG	TCAA GTCTGCAGGCCAGATGAGGG
		GGCC CCCTCATCTGGCCTGCAGAC
hVEGFA-site3	ATTCATTGATCCGGGTTTTA	TCAA ATTCATTGATCCGGGTTTTA
		GGCC TAAAACCCGGATCAATGAAT
hVEGFA-site4	CCGGGTTTTATCCCTCTTCT	TCAA CCGGGTTTTATCCCTCTTCT
		GGCC AGAAGAGGGATAAAACCCGG
hVEGFA-site5	CCTTATATTCCTGTGCCCCT	TCAA CCTTATATTCCTGTGCCCCT
		GGCC AGGGGCACAGGAATATAAGG
hVEGFA-site6	TAGTCATCTTCTCCCCTATC	TCAA TAGTCATCTTCTCCCCTATC
		GGCC GATAGGGGAGAAGATGACTA
hVEGFA-site7	GATGCTTTGCCGTAACCCTT	TCAA GATGCTTTGCCGTAACCCTT
		GGCC AAGGGTTACGGCAAAGCATC
hVEGFA-site8	TGGGAAGAAGGTGGGGAGAA	TCAA TGGGAAGAAGGTGGGGAGAA
		GGCC TTCTCCCCACCTTCTTCCCA

hVEGFA-site9	GTCAGCTAATTCTGACTCCT	TCAA GTCAGCTAATTCTGACTCCT
		GGCC AGGAGTCAGAATTAGCTGAC
hVEGFA-site10	GGTGGAAAGCTTAGGGAAGT	TCAA GGTGGAAAGCTTAGGGAAGT
		GGCC ACTTCCCTAAGCTTTCCACC
hVEGFA-site11	CCGCATAATCTGGAAAGGAA	TCAA CCGCATAATCTGGAAAGGAA
		GGCC TTCCTTTCCAGATTATGCGG
Hpd-site1	CAGGCTTATGGAAACTGTGA	TCAA GACGTGGCTGACTACCTCCC
		GGCC GGGAGGTAGTCAGCCACGTC
Hpd-site2	CATAAGCCTGAAAATGTCTC	TCAA CTTCTCCACCAGGGTGTGTG
		GGCC CACACACCCTGGTGGAGAAG
Hpd-site3	TTGAAGGCCCAAGTGAAGCC	TCAA GGATTCCTCGTAGTTGGTCA
		GGCC TGACCAACTACGAGGAATCC
Hpd-site4	GGGCATTTTGATGGATTCCT	TCAA GGGCATTTTGATGGATTCCT
		GGCC AGGAATCCATCAAAATGCCC
Tyr-site1	TGAAGGCCCAAGTGAAGCCC	TCAA TAGAAGAAACATTTTTGATT
		GGCC AATCAAAAATGTTTCTTCTA
Tyr-site2	GAAGGCCCAAGTGAAGCCCT	TCAA TTGAGTGTCTCCGAAAAGAA
		GGCC TTCTTTTCGGAGACACTCAA
Tyr-site3	AAGGCCCAAGTGAAGCCCTC	TCAA ATCATTAAACATGGGTGTTG
		GGCC CAACACCCATGTTTAATGAT
Tyr-site4	GTCCCTTCTCAAAAAACTTA	TCAA TTTGCCCATGAAGCACCAGG
		GGCC CCTGGTGCTTCATGGGCAAA
Pcsk9-site1	TCCCTTCTCAAAAAACTTAC	TCAA TGATCAGGCGAGCAAGTGTG
		GGCC CACACTTGCTCGCCTGATCA
Pcsk9-site2	TCATCTTTTGTCCCTTCTCA	TCAA GACGTCTTTGGTAGAGAAGT
		GGCC ACTTCTCTACCAAAGACGTC
Pcsk9-site3	GTCATCTTTTGTCCCTTCTC	TCAA TTTGCATTCCAGCCCTGGGG
		GGCC CCCCAGGGCTGGAATGCAAA
Klkb1-site1	AGAAGGGACAAAAGATGACA	TCAA GGCCACACTGCTTTAAAGAA
		GGCC TTCTTTAAAGCAGTGTGGCC
Klkb1-site2	CTTCCACCCGGATAAGATGC	TCAA ATGAGAGGGTCCAACTTTAA
		GGCC TTAAAGTTGGACCCTCTCAT
Klkb1-site3	TATCCCGAGTATCTGGAAGA	TCAA TGCTTCATAGGTGAAACGCA
		GGCC TGCGTTTCACCTATGAAGCA
Dmd-site1	ATATACTTTTTCTTCCAAAT	TCAA ATATACTTTTTCTTCCAAAT
		GGCC ATTTGGAAGAAAAAGTATAT
Dmd-site2	TTGGAATATAATCCTCCACT	TCAA TTGGAATATAATCCTCCACT
		GGCC AGTGGAGGATTATATTCCAA
Dmd-site3	GACGTTAACCTGTGGATAAT	TCAA GACGTTAACCTGTGGATAAT

		GGCC ATTATCCACAGGTTAACGTC
Dmd-site4	TGGAATAGTGTGGTTTCACA	TCAA TGGAATAGTGTGGTTTCACA
		GGCC TGTGAAACCACACTATTCCA
Dmd-site5	CCTGAAGGTTGGTAGATTCT	TCAA CCTGAAGGTTGGTAGATTCT
		GGCC AGAATCTACCAACCTTCAGG
Dmd-site6	GGAGACGGAAGTAAATCTGG	TCAA GGAGACGGAAGTAAATCTGG
		GGCC CCAGATTTACTTCCGTCTCC
Dmd-site7	GAGATGTCAGATCCATCATG	TCAA GAGATGTCAGATCCATCATG
		GGCC CATGATGGATCTGACATCTC
Dmd-site8	GAATCCAGCGGTGATCATGC	TCAA GAATCCAGCGGTGATCATGC
		GGCC GCATGATCACCGCTGGATTC
Dmd-site9	TCTTTCATCCTCAGGTACTG	TCAA TCTTTCATCCTCAGGTACTG
		GGCC CAGTACCTGAGGATGAAAGA
Dmd-site10	CTTTAAAGCCACTTGTCTGA	TCAA CTTTAAAGCCACTTGTCTGA
		GGCC TCAGACAAGTGGCTTTAAAG
Dmd-site11	TGAGTGAACTTAGTTTTTCC	TCAA TGAGTGAACTTAGTTTTTCC
		GGCC GGAAAAACTAAGTTCACTCA
Dmd-site12	GCACTCACCTTTTCCTGAGT	TCAA GCACTCACCTTTTCCTGAGT
		GGCC ACTCAGGAAAAGGTGAGTGC
Dmd-site13	ACTCTAGCCAGTTAACTCTC	TCAA ACTCTAGCCAGTTAACTCTC
		GGCC GAGAGTTAACTGGCTAGAGT
Dmd-site14	GGTGTGAGGGCCAAAGAGAA	TCAA GGTGTGAGGGCCAAAGAGAA
		GGCC TTCTCTTTGGCCCTCACACC
Dmd-site15	GTTTTCCTGAAAGAGGAATG	TCAA GTTTTCCTGAAAGAGGAATG
		GGCC CATTCCTCTTTCAGGAAAAC
Dmd-site16	CAGTTCATCCATGACTCCTC	TCAA CAGTTCATCCATGACTCCTC
		GGCC GAGGAGTCATGGATGAACTG
Dmd-site17	GTTGCACAGGTATGTTTTAT	TCAA GTTGCACAGGTATGTTTTAT
		GGCC ATAAAACATACCTGTGCAAC
Dmd-site18	GAACGAGTAACAGCTTTGAA	TCAA GAACGAGTAACAGCTTTGAA
		GGCC TTCAAAGCTGTTACTCGTTC
Dmd-site19	CAGAACATAGAACAAATCAC	TCAA CAGAACATAGAACAAATCAC
		GGCC GTGATTTGTTCTATGTTCTG
Dmd-site20	GGCAAACCGCGGTGACCACT	TCAA GGCAAACCGCGGTGACCACT
		GGCC AGTGGTCACCGCGGTTTGCC
Dmd-site21	TTTGCTCAATAGGAAATTGA	TCAA TTTGCTCAATAGGAAATTGA
		GGCC TCAATTTCCTATTGAGCAAA
Dmd-site22	CGTGAATTGCAGAAGAAGAA	TCAA CGTGAATTGCAGAAGAAGAA

		GGCC TTCTTCTTCTGCAATTCACG
Dmd-site23	CATCTTCTAAATACTCCTGA	TCAA CATCTTCTAAATACTCCTGA
		GGCC TCAGGAGTATTTAGAAGATG
Dmd-site24	ATTATTCACAAGAAGAAGAC	TCAA ATTATTCACAAGAAGAAGAC
		GGCC GTCTTCTTCTTGTGAATAAT
Dmd-site25	TAAATACCTTCATATCATAA	TCAA TAAATACCTTCATATCATAA
		GGCC TTATGATATGAAGGTATTTA
Dmd-site26	CGAGTTATAAAATCACAGAG	TCAA CGAGTTATAAAATCACAGAG
		GGCC CTCTGTGATTTTATAACTCG
Dmd-site27	GTCTTCCAGATCACCCACCA	TCAA GTCTTCCAGATCACCCACCA
		GGCC TGGTGGGTGATCTGGAAGAC
Dmd-site28	GATCATTTCATTGATGTCTT	TCAA GATCATTTCATTGATGTCTT
		GGCC AAGACATCAATGAAATGATC
Dmd-site29	TGCTGGTTTTGTTTTTCAAA	TCAA TGCTGGTTTTGTTTTTCAAA
		GGCC TTTGAAAAACAAAACCAGCA
Dmd-site30	ATTCTCTGTTATCATGTGTA	TCAA ATTCTCTGTTATCATGTGTA
		GGCC TACACATGATAACAGAGAAT
Dmd-site31	GAAAATGGCCAAAAAATCCT	TCAA GAAAATGGCCAAAAAATCCT
		GGCC AGGATTTTTTGGCCATTTTC
Dmd-site32	CACCTCAGCTTGGCGCAACT	TCAA CACCTCAGCTTGGCGCAACT
		GGCC AGTTGCGCCAAGCTGAGGTG
Dmd-site33	ATAGTAGGGCACTTTGTTTG	TCAA ATAGTAGGGCACTTTGTTTG
		GGCC CAAACAAAGTGCCCTACTAT
Dmd-site34	GCTTGGCAGTTTCAGCAGCA	TCAA GCTTGGCAGTTTCAGCAGCA
		GGCC TGCTGCTGAAACTGCCAAGC
Dmd-site35	CAGAGTAACGGGACTGCAAA	TCAA CAGAGTAACGGGACTGCAAA
		GGCC TTTGCAGTCCCGTTACTCTG
Dmd-site36	TTTATTTTCCAGAGATGATG	TCAA TTTATTTTCCAGAGATGATG
		GGCC CATCATCTCTGGAAAATAAA
Dmd-site37	TTCCTTAGAGAGTGAGGAAA	TCAA TTCCTTAGAGAGTGAGGAAA
		GGCC TTTCCTCACTCTCTAAGGAA

Supplementary Table S3. PCR and IVT primers used in this study.

Primer	Primer sequences (5'-3')
lzf256-mTyr -1f	AACAGGCTGAGAGTATTTGATGT
lzf257-mTyr -1r	CTATATAGTGCATCTTACCTGCC
lzf258-mTyr -2f	GTTGCTGGAAAAGAAGTCTG
lzf259-mTyr -2r	CTCATCTGTGCAAATGTCAC
lzf346-mPcsk9-Exon4-1f	TCAGTTTACCTCCTGGTTCTGTC
lzf347-mPcsk9-Exon4-1r	ACATGTGACAACACTGTAAGAGC

lzf348-mPcsk9-Exon4-2f	CATGAGCCGTCTAATGCGTG
lzf349-mPcsk9-Exon4-2r	TCAGTTTCCCACCTGCATTC
lzf358-mPcsk9-Exon9-1f	GAGCGTTAGTTGGGACCAGAAAG
lzf359-mPcsk9-Exon9-1r	GCCTGCCATACACAAATGCACAC
lzf360-mPcsk9-Exon9-2f	TACAGAGTCTGAGCTGCATG
lzf361-mPcsk9-Exon9-2r	GCTACCCTGACACATGGACC
lzf1121_mHpd-exon3-1f	CAATCAGGGTCCCCAAGGACCTT
lzf1122_mHpd-exon3-1r	GAGAAGTTTGAAACCAGGAAGAT
lzf1123_mHpd-exon3-2f	AGAGTCTCCAAATGACGGAC
lzf1124_mHpd-exon3-2r	TACATCTTGGAACCAGCTAG
lzf1127_mHpd-exon7-1f	CTGAGTTAGGGTCAGCTTCATGG
lzf1128_mHpd-exon7-1r	AAATGACGGAGCTGCCTGTGAAC
lzf1129_mHpd-exon7-2f	TAGAGAAGAGTGGGGGCTTT
lzf1130_mHpd-exon7-2r	GTTTCCCACCAGATGCTTAC
lzf1135_mHpd-exon9-1f	TGAGGATCCTGTGTAACGGGTGT
lzf1136_mHpd-exon9-1r	GTTTGTGGGAGAGGAAAGGGACG
lzf1137_mHpd-exon9-2f	GAAGAGGGTGGGAAGGTCTC
lzf1138_mHpd-exon9-2r	CGCTACTCTCATCGGCAGAG
lzf2045_mDmd-sgRNA1-1f	CTTGAAGGCAATAGCCTTTATAG
lzf2046_mDmd-sgRNA1-1r	GATAATAAAGTAGATAAATGACG
lzf2047_mDmd-sgRNA1-2f	GAAGTTTATTGGCTTCTCAT
lzf2048_mDmd-sgRNA1-2r	TGTAATCAATCTGCCTACTC
lzf2051_mDmd-sgRNA2-1f	CAATTAGTTATTTTCTATCTATT
lzf2052_mDmd-sgRNA2-1r	TAGTCCTAGAAAGATGGTTAGAT
lzf2053_mDmd-sgRNA2-2f	TAGTGAATATAGGAAGCACT
lzf2054_mDmd-sgRNA2-2r	GATATATTAATGATATTGGT
lzf2057_mDmd-sgRNA3-1f	TCTTATTAAAGCATGACAGATGC
lzf2058_mDmd-sgRNA3-1r	TAACAGCATGCAGCCTAGTAGAG
lzf2059_mDmd-sgRNA3-2f	CTATCATGGCTGGATTGCAG
lzf2060_mDmd-sgRNA3-2r	ATTAAATCTCAAAATAAATG
lzf2063_mDmd-sgRNA4-1f	CTCAATTCAGAATTGGAATGGAT
lzf2064_mDmd-sgRNA4-1r	GTATTATCAGAACACAGGAAAAC
lzf2065_mDmd-sgRNA4-2f	GTGTATGTGTTTGTTTCAGG
lzf2066_mDmd-sgRNA4-2r	CTCATTCTACACAATTTATT
lzf2071_mDmd-sgRNA6-1f	ATATTTCATTCCATCTCTCATTT
lzf2072_mDmd-sgRNA6-1r	TACCTCATGAGCATGAAACTGTT
lzf2073_mDmd-sgRNA6-2f	ACCACTAATTGTATACCACC
lzf2074_mDmd-sgRNA6-2r	CACTTCTTCAACATCATTTG
lzf2077_mDmd-sgRNA7-1f	TAAGATATGCTTAAGAAGAATAT
lzf2078_mDmd-sgRNA7-1r	ATGCTAGCTACCCTGAGACATTC
lzf2079_mDmd-sgRNA7-2f	AATTGCAACTAATAAAATTC
lzf2080_mDmd-sgRNA7-2r	CAAATGAATCTCCTAAATTC
lzf2083_mDmd-sgRNA8-1f	TGAGCAATTGCATTACCTTATAT

lzf2084_mDmd-sgRNA8-1r	GAAAGTGATACTGCACAAGTGGC
lzf2085_mDmd-sgRNA8-2f	TCAGGTGCTTCAAGAAGATC
lzf2086_mDmd-sgRNA8-2r	TAGATACATTTTCATATTGG
lzf2089_mDmd-sgRNA9-1f	GTGGAAGAATGACTGGATTAATC
lzf2090_mDmd-sgRNA9-1r	CAATGAATAAGTGATTTAAGATA
lzf2091_mDmd-sgRNA9-2f	CTATTCTTTACAGGAGATCC
lzf2092_mDmd-sgRNA9-2r	GTTAAATAATAATTGTACAC
lzf2097_mDmd-sgRNA11-1f	ACACAATTAAAGGAGATTGAATT
lzf2098_mDmd-sgRNA11-1r	TAAGAATTAATATCACTTACTTG
lzf2099_mDmd-sgRNA11-2f	ACCTAGACTTAATTTCATTC
lzf3000_mDmd-sgRNA11-2r	GTCCCAACGTTGTGCAAAGT
lzf3003_mDmd-sgRNA12-1f	TCACAGATTTCACAGGCTGTCAC
lzf3004_mDmd-sgRNA12-1r	GCATAATGATTTCTTGGGTAAAT
lzf3005_mDmd-sgRNA12-2f	ACGGTAACTATGGTGACCAC
lzf3006_mDmd-sgRNA12-2r	TTGGGAAATGTGATTCAACT
lzf3009_mDmd-sgRNA13-1f	TCTTCATGGGATATGTATTTTGG
lzf3010_mDmd-sgRNA13-1r	GAAGGAGGAAAACCTTACCTTAC
lzf3011_mDmd-sgRNA13-2f	TGTAGAGGGTGTTAATGCTG
lzf3012_mDmd-sgRNA13-2r	TGGTAGACTGGGTTTTCAAC
lzf3015_mDmd-sgRNA14-1f	GATGAAGTCAACAGATTGTCAGC
lzf3016_mDmd-sgRNA14-1r	CTCTAGCTGCAAATGTAGCTTGT
lzf3017_mDmd-sgRNA14-2f	TCAGCCTCAAATTGAGCAAT
lzf3018_mDmd-sgRNA14-2r	CTTGGTCCTGGGAAGGCTAG
lzf3021_mDmd-sgRNA15-1f	GATATATCATCATGATGAACTAT
lzf3022_mDmd-sgRNA15-1r	CAGGTACTGGAAACTGTCCTAGC
lzf3023_mDmd-sgRNA15-2f	TTCCAGAATCACATAAAAAC
lzf3024_mDmd-sgRNA15-2r	GGCTGAGCTAATTATATAAT
lzf3027_mDmd-sgRNA16-1f	TCAGTCTCTTGAAAATCTGATGC
lzf3028_mDmd-sgRNA16-1r	TCAGACTAAACTGAGCAGAAATC
lzf3029_mDmd-sgRNA16-2f	CAAATCAGATTCGTCTATTG
lzf3030_mDmd-sgRNA16-2r	AGACTGTGTCACTCATATAT
lzf3033_mDmd-sgRNA17-1f	GCAATAATATTAAGAGTATGAAT
lzf3034_mDmd-sgRNA17-1r	TAAATGTTATACTAAGCAGTCGT
lzf3035_mDmd-sgRNA17-2f	TCCAATCAGATTTGACAAGT
lzf3036_mDmd-sgRNA17-2r	TACAGTGTACAGAAGTTATT
lzf3039_mDmd-sgRNA18-1f	CTGAGTGAAGTCAAGTCTGAAGT
lzf3040_mDmd-sgRNA18-1r	CTGAGAATCACAATAAGGGTTTC
lzf3041_mDmd-sgRNA18-2f	CCGGACGTCAAATTGTACAG
lzf3042_mDmd-sgRNA18-2r	TCTATTTTATCTTGAATACT
lzf3045_mDmd-sgRNA19-1f	TGTAGATAGTTGAACAAATGTTT
lzf3046_mDmd-sgRNA19-1r	TTAAAACATTATTTTCATAACAG
lzf3047_mDmd-sgRNA19-2f	ATAACATGGTATATTTCCAT
lzf3048_mDmd-sgRNA19-2r	TACTTCTCATATAATTTCAT

lzf3051_mDmd-sgRNA20-1f	TTCTTGCTCATGGAATATAGCGT
lzf3052_mDmd-sgRNA20-1r	CTTCAGAGTATTGCGCAACCTTC
lzf3053_mDmd-sgRNA20-2f	TAAAGGCTGAAATGAATGAC
lzf3054_mDmd-sgRNA20-2r	ACAAGTTTCCACCTTGAAGT
lzf3057_mDmd-sgRNA21-1f	GCTTATTGGGTGAGGATGACAGT
lzf3058_mDmd-sgRNA21-1r	CACAATTTGTGCAAAGTTGAGTC
lzf3059_mDmd-sgRNA21-2f	CAGCCTATGAAAGTTCTGAG
lzf3060_mDmd-sgRNA21-2r	TGCTGAGCTGGATCTGAGTT
lzf3065_mDmd-sgRNA23-1f	CAGCACACTCTCCATGAAGAAAC
lzf3066_mDmd-sgRNA23-1r	TAGTTTATACATTACCTACCAAG
lzf3067_mDmd-sgRNA23-2f	GTAGTGACGACTGAAGATAT
lzf3068_mDmd-sgRNA23-2r	ATTCTTCAATGTGCAGTAAC
lzf3071_mDmd-sgRNA24-1f	TAAGGTTGATAATTTAGAATTGT
lzf3072_mDmd-sgRNA24-1r	TAATGTTTCAGTAACATTAAAAG
lzf3073_mDmd-sgRNA24-2f	TACTCCCTAGAGAAAGCTAG
lzf3074_mDmd-sgRNA24-2r	GGCTACTTACCCTTGTCGTT
lzf3077_mDmd-sgRNA25-1f	TACTACAAAAGTAATACCTTTGT
lzf3078_mDmd-sgRNA25-1r	TATTGCTGAAAAAATGAAGCCAG
lzf3079_mDmd-sgRNA25-2f	GTGTCCTATAAACCACTTAC
lzf3080_mDmd-sgRNA25-2r	GTCTTACCTTAAGATACCAT
lzf3087_mDmd-sgRNA28-1f	TGACACAATCTGTGGTTACTAAG
lzf3088_mDmd-sgRNA28-1r	AGGTAGCCTAAAAACTATTAGTC
lzf3089_mDmd-sgRNA28-2f	ATGCCATCTTCTTTGCTGTT
lzf3090_mDmd-sgRNA28-2r	GAAATGGAAAGTGACAATAT
lzf3093_mDmd-sgRNA29-1f	CATATTTTCATTTCTAAAAGTCT
lzf3094_mDmd-sgRNA29-1r	TTCTTCAGTAAAATGGCTATCAT
lzf3095_mDmd-sgRNA29-2f	TTCAGGCAACACTGCAAGAT
lzf3096_mDmd-sgRNA29-2r	CTATACCTTGAGCTGTTACT
lzf3099_mDmd-sgRNA30-1f	GATGCACCGTTTAAAGATGTCTG
lzf3100_mDmd-sgRNA30-1r	CAGGACAGCAAGCCAGGCTTTTG
lzf3101_mDmd-sgRNA30-2f	CGGCAGATAAGTGTAGACGT
lzf3102_mDmd-sgRNA30-2r	CATGGTTCATCCAAGGTCAC
lzf3105_mDmd-sgRNA31-1f	ATTCACATTCCCAGCAAGTCTCT
lzf3106_mDmd-sgRNA31-1r	GCCATCTTTATTCATATCTGGAT
lzf3107_mDmd-sgRNA31-2f	TGGTAATTCTGAATGTGTTT
lzf3108_mDmd-sgRNA31-2r	TCTGAAGTTCACTCCACTTG
lzf3111_mDmd-sgRNA32-1f	GAAAGCAGGCTGAAGAGGTCAAC
lzf3112_mDmd-sgRNA32-1r	TGAGTACTCTTTTGGGATCTCTC
lzf3113_mDmd-sgRNA32-2f	GACAAATTGAACCTGCGCTC
lzf3114_mDmd-sgRNA32-2r	CAAAACAAAGCACACAGTAC
lzf3117_mDmd-sgRNA33-1f	TGTAATTCTGGAGATTAATGTTG
lzf3118_mDmd-sgRNA33-1r	TATGAATTTATTATCTATGCTTC
lzf3119_mDmd-sgRNA33-2f	TCCTGTTTACCACGAATTTG

lzf3120_mDmd-sgRNA33-2r	TAGATATTGTAGATGAGAAT
lzf3123_mDmd-sgRNA34-1f	CTTTGCATACTATTGTCCCACTT
lzf3124_mDmd-sgRNA34-1r	GAAAGGAACAAACTCACAGCAAC
lzf3125_mDmd-sgRNA34-2f	CAATAATAAACCTGAGATTG
lzf3126_mDmd-sgRNA34-2r	AACCTAGTCACGACAAATTG
lzf3129_mDmd-sgRNA35-1f	CATTCACGGTTTACTTTTAGTTG
lzf3130_mDmd-sgRNA35-1r	TAAATTGGAAATTAAAATGTCAT
lzf3131_mDmd-sgRNA35-2f	CCTTTTGACCTTTCCATAAT
lzf3132_mDmd-sgRNA35-2r	AGCTGAATAAACAAACAAAG
lzf3137_mDmd-sgRNA36-1f	TGTCTTTAGAATAGGGGAACAAT
lzf3138_mDmd-sgRNA36-1r	ACAGATTATTTTATCAAACAGTT
lzf3139_mDmd-sgRNA36-2f	TGAGAGCAAAGAAATGTTTC
lzf3140_mDmd-sgRNA36-2r	TTCTCCATGTGCAAGTGTGT
lzf4303-mKlkb1-exon3-1f	GGACTTGTCGCAAGAACGTTCTC
lzf4304-mKlkb1-exon3-1r	TTATCTTTCTTGGTGGTCTCGTC
lzf4305-mKlkb1-exon4-1f	CAGTGTTTGATAATTTAGACATG
lzf4306-mKlkb1-exon4-1r	GTTGACTACAGGGAGTTTGCTAC
lzf4311-mKlkb1-exon13-1f	CGGATCACTGCTCCTTCATCTCC
lzf4312-mKlkb1-exon13-1r	TCGTTTTTGAATGAACTGTCTTC
lzf2041_ZF709-IVT-1f	GAAAT TAATACGACTCACTATAGG G GATTCAAGAATCCCGAAGT
lzf2042_ZF709-IVT-1r	TTCTTTTCGGAGACACTCAA TTGAACCTCACACGACTAAA
lzf3141_ZF759- IVT-1r	ATTATCCACAGGTTAACGTC TTGAACCTCACACGACTAAA
lzf3142_ZF769- IVT-1r	GAGAGTTAACTGGCTAGAGT TTGAACCTCACACGACTAAA
lzf3143_ZF774- IVT-1r	TTCAAAGCTGTTACTCGTTC TTGAACCTCACACGACTAAA
lzf3144_ZF780- IVT-1r	GTCTTCTTCTTGTGAATAAT TTGAACCTCACACGACTAAA
lzf3145_ZF789- IVT-1r	CAAACAAAGTGCCCTACTAT TTGAACCTCACACGACTAAA
lzf5034_TnpB-IVT-1f	GAAAT TAATACGACTCACTATAGG G tGGTGGCTGCGGGAATCTC
lzf5035_ IVT-mKlkb1-sg1-1r	TTCTTTAAAGCAGTGTGGCC TTGAACCTCACACGACTAAA
lzf5036_ IVT-mKlkb1-sg2-1r	TTAAAGTTGGACCCTCTCAT TTGAACCTCACACGACTAAA
lzf5037_ IVT-mKlkb1-sg3-1r	TGCGTTTCACCTATGAAGCA TTGAACCTCACACGACTAAA

Supplementary Table S4. NGS primers used in this study.

lzf3762_ZF704-R701-1f	GAATTC NNNNNN GGATCC TCCAGACTGGTCCCCACAAC
lzf3769_ZF704-R707-1r	GACACACTGGTGCCCTCATC
lzf3803_ZF709-R701-1f	GAATTC NNNNNN GGATCC CAACTGCGGAAACTGTAAGT
lzf3832_ZF709-R731-1r	CATGGGTGTTGACCCATTGT
lzf3833_ZF759-R701-1f	GAATTC NNNNNN GGATCC GAAAACTATCATGGCTGGAT
lzf4008_mDmd-exon6-2r	TGTACCTGTGACTATGGATAAG
lzf3855_ZF769-R701-1f	GAATTC NNNNNN GGATCC TATTTTGGTTTTCTTTGTAG
lzf3884_ZF769-R731-1r	TTTTCGGCAGTAGTTGTCAT
lzf3885_ZF774-R701-1f	GAATTC NNNNNN GGATCC GGTGATTAAAACCGGACGTC
lzf3643_mDmd-exon33-2r	CTGGTATTCTATTTTATCTT
lzf3904_ZF780-R701-1f	GAATTC NNNNNN GGATCC GCTAGTTTACTTTATGATAT
lzf3647_mDmd-exon43-2r	GTACATTCTATGAAGTTTTT
lzf3924_ZF789-R701-1f	GAATTC NNNNNN GGATCC TCTGGAGATTAATGTTGCCT
lzf3960_ZF789-R738-1r	TTCATGAACATACAGATCAG
lzf3961_ZF790-R701-1f	GAATTC NNNNNN GGATCC CTCCTTCTGTTTTCCCAGGC
lzf3985_ZF790-R726-1r	GAATACTAATACCTGAATCC
lzf3986_ZF759-R701-1f	GAATTC NNNNNN GGATCC GTTATATTTTAACATATAGGTC
lzf4672_ZF1162-R715-1f	GAATTC NNNNNN GGATCC TGTGTTTGTTCAATTCCGCT
lzf4675_ZF1162-R717-1r	GTCCATCATAGTATAAACTT
lzf4676_ZF1163-R718-1f	GAATTC NNNNNN GGATCC GAAGAGCAGTTTATTTGTGT
lzf4661_ZF1163-R705-1r	CTGTAAAATGCACTTGTAGC
lzf4662_ZF1164-R706-1f	GAATTC NNNNNN GGATCC CCTCTCCCGGTCTCTCTTCT
lzf4663_ZF1164-R706-1r	GTTTATAACATAATCTCTGT
lzf5038_ZF1699-R708-1f	GAATTC NNNNNN GGATCC GTGGTGCTTAGAGAAGAGTG
lzf5040_ZF1699-R709-1r	CATCAAGGGACACTCACAGT
lzf5042_ZF1725-R711-1f	GAATTC NNNNNN GGATCC TGAAAAACCTGCAGTTCCAC
lzf5043_ZF1700-R711-1r	GCTCTCTCAGAGCCCGCAAG
lzf5045_ZF1726-R713-1f	GAATTC NNNNNN GGATCC

	TGAAAAACCTGCAGTTCCAC
lzf5046_ZF1701-R713-1r	GCTCTCTCAGAGCCCGCAAG
lzf5048_ZF1727-R715-1f	GAATTC NNNNNN GGATCC CTTCATGGGTTTCAACTGCG
lzf5049_ZF1702-R715-1r	TGACCCATTGTTCATTTGGC
lzf5051_ZF1729-R719-1f	GAATTC NNNNNN GGATCC CTGCGGAAACTGTAAGTTTG
lzf5052_ZF1704-R719-1r	CATGGGTGTTGACCCATTGT
lzf5054_ZF1730-R721-1f	GAATTC NNNNNN GGATCC CCTCTTTGTATGGATGCATT
lzf5055_ZF1705-R721-1r	TTTTCTGCATCTCTCCAATC

Supplementary Table S5. **Human 14 target sites sequences and oligos for TnpB activity evaluation in this study.**

sgRNA	Target site sequences (5'-3')	oligos
Site1	TTTACACATCATCATATACA	TCAA TTTACACATCATCATATACA
		GGCC TGTATATGATGATGTGTAAA
Site2	AGAAGTGAGATGGCTCCAAA	TCAA AGAAGTGAGATGGCTCCAAA
		GGCC TTTGGAGCCATCTCACTTCT
Site3	GACCCAAAGAAATGTATTCC	TCAA GACCCAAAGAAATGTATTCC
		GGCC GGAATACATTTCTTTGGGTC
Site4	ATTCAAAAACACGCAAACCC	TCAA ATTCAAAAACACGCAAACCC
		GGCC GGGTTTGCGTGTTTTTGAAT
Site5	TCCTTTGCCAGGTTTCTGCA	TCAA TCCTTTGCCAGGTTTCTGCA
		GGCC TGCAGAAACCTGGCAAAGGA
Site6	TAATTAGAGCATAAATAAGA	TCAA TAATTAGAGCATAAATAAGA
		GGCC TCTTATTTATGCTCTAATTA
Site7	TCAAGTACCACCAGTTTTAT	TCAA TCAAGTACCACCAGTTTTAT
		GGCC ATAAAACTGGTGGTACTTGA
Site8	AGAACACCCATAAGAACAAC	TCAA AGAACACCCATAAGAACAAC
		GGCC GTTGTTCTTATGGGTGTTCT
Site9	GAAAAGATTACAGAATCAGG	TCAA GAAAAGATTACAGAATCAGG
		GGCC CCTGATTCTGTAATCTTTTC
Site10	AGATGATGTTTCCACACATA	TCAA AGATGATGTTTCCACACATA
		GGCC TATGTGTGGAAACATCATCT
Site11	CTGCCTTCAGAAAGCACCTT	TCAA CTGCCTTCAGAAAGCACCTT
		GGCC AAGGTGCTTTCTGAAGGCAG
Site12	AAAAATGCATGAAGCTCCTT	TCAA AAAAATGCATGAAGCTCCTT
		GGCC AAGGAGCTTCATGCATTTTT
Site13	TAAGGAACTAGAATCTAAAA	TCAA TAAGGAACTAGAATCTAAAA
		GGCC TTTTAGATTCTAGTTCCTTA
Site14	GAGTCCAGTCAGAAAGCAGA	TCAA GAGTCCAGTCAGAAAGCAGA

		GGCC TCTGCTTTCTGACTGGACTC

Supplementary Table S6. SaCas9 target site sequences in this study.

sgRNA	Target site sequences (5'-3')	oligos
Tyr-site1	GTCTTGATTAGAAGAAACATT	CACC G GTCTTGATTAGAAGAAACATT
		TAAC AATGTTTCTTCTAATCAAGAC C
Tyr-site2	ATTAGAAGAAACATTTTTGAT	CACC G ATTAGAAGAAACATTTTTGAT
		TAAC ATCAAAAATGTTTCTTCTAATC
Tyr-site3	ACTCAAATCAAAAATGTTTCT	CACC G ACTCAAATCAAAAATGTTTCT
		TAAC AGAAACATTTTTGATTTGAGT C
Tyr-site4	TTTGATTTGAGTGTCTCCGAA	CACC G TTTGATTTGAGTGTCTCCGAA
		TAAC TTCGGAGACACTCAAATCAAA C
Tyr-site5	ATTTGAGTGTCTCCGAAAAGA	CACC G ATTTGAGTGTCTCCGAAAAGA
		TAAC TCTTTTCGGAGACACTCAAAT C
Tyr-site6	AACAATGGGTCAACACCCATG	CACC G AACAATGGGTCAACACCCATG
		TAAC CATGGGTGTTGACCCATTGTT C
Tyr-site7	AATGGGTCAACACCCATGTTT	CACC G AATGGGTCAACACCCATGTTT
		TAAC AAACATGGGTGTTGACCCATTC
Tyr-site8	TAGATGTTGATATCATTAAAC	CACC G TAGATGTTGATATCATTAAAC
		TAAC GTTTAATGATATCAACATCTAC
Tyr-site9	TTGATTTTGCCCATGAAGCAC	CACC G TTGATTTTGCCCATGAAGCAC
		TAAC GTGCTTCATGGGCAAAATCAAC
Tyr-site10	GAAACCCTGGTGCTTCATGGG	CACC G GAAACCCTGGTGCTTCATGGG
		TAAC CCCATGAAGCACCAGGGTTTCC
Klkb1-site1	ACACTGCTTTAAAGAATGACC	CACC G ACACTGCTTTAAAGAATGACC
		TAAC GGTCATTCTTTAAAGCAGTGTC

Klkb1-site2	TTTGATGGCCACACTGCTTTA	CACC G TTTGATGGCCACACTGCTTTA
		TAAC TAAAGCAGTGTGGCCATCAAAC
Klkb1-site3	TTAAAGTTGGACCCTCTCATA	CACC G TTAAAGTTGGACCCTCTCATA
		TAAC TATGAGAGGGTCCAACTTTAAC
Klkb1-site4	CTTGATATGAGAGGGTCCAAC	CACC G CTTGATATGAGAGGGTCCAAC
		TAAC GTTGGACCCTCTCATATCAAGC
HEK1-site5	GTTTCACCTATGAAGCAATCA	CACC G GTTTCACCTATGAAGCAATCA
		TAAC TGATTGCTTCATAGGTGAAACC
Klkb1-site6	TATTTTGCGTTTCACCTATGA	CACC G TATTTTGCGTTTCACCTATGA
		TAAC TCATAGGTGAAACGCAAAATAC
Klkb1-site7	GATTGCTTCATAGGTGAAACG	CACC G GATTGCTTCATAGGTGAAACG
		TAAC CGTTTCACCTATGAAGCAATCC
Hpd-site1	GTGGCTGACTACCTCCCGGGA	CACC G GTGGCTGACTACCTCCCGGGA
		TAAC TCCCGGGAGGTAGTCAGCCACC
Hpd-site2	CCTAGAGACTGGCTCCCGGGA	CACC G CCTAGAGACTGGCTCCCGGGA
		TAAC TCCCGGGAGCCAGTCTCTAGGC
Hpd-site3	GATCTTCTCCACCAGGGTGTG	CACC G GATCTTCTCCACCAGGGTGTG
		TAAC CACACCCTGGTGGAGAAGATCC
Hpd-site4	AGTGTAGTTGATCTTCTCCAC	CACC G AGTGTAGTTGATCTTCTCCAC
		TAAC GTGGAGAAGATCAACTACACTC
Hpd-site5	TACCACACACACCCTGGTGGA	CACC G TACCACACACACCCTGGTGGA
		TAAC TCCACCAGGGTGTGTGTGGTAC
Hpd-site6	GGATTCCTCGTAGTTGGTCAC	CACC G

		GGATTCCTCGTAGTTGGTCAC
		TAAC GTGACCAACTACGAGGAATCCC
Hpd-site7	CATTTTGATGGATTCCTCGTA	CACC G CATTTTGATGGATTCCTCGTA
		TAAC TACGAGGAATCCATCAAAATGC
Hpd-site8	GACCAACTACGAGGAATCCAT	CACC G GACCAACTACGAGGAATCCAT
		TAAC ATGGATTCCTCGTAGTTGGTCC
Hpd-site9	GGCTCGTTGATGGGCATTTTG	CACC G GGCTCGTTGATGGGCATTTTG
		TAAC CAAAATGCCCATCAACGAGCCC
Pcsk9-site1	GACATAGCCCCGGCGGGCAGC	CACC G GACATAGCCCCGGCGGGCAGC
		TAAC GCTGCCCGCCGGGGCTATGTCC
Pcsk9-site2	TGCCCGCCGGGGCTATGTCAT	CACC G TGCCCGCCGGGGCTATGTCAT
		TAAC ATGACATAGCCCCGGCGGGCAC
Pcsk9-site3	CGTGGCTGTCACACTTGCTCG	CACC G CGTGGCTGTCACACTTGCTCG
		TAAC CGAGCAAGTGTGACAGCCACGC
Pcsk9-site4	GATGACGTCTTTGGTAGAGAA	CACC G GATGACGTCTTTGGTAGAGAA
		TAAC TTCTCTACCAAAGACGTCATCC
Pcsk9-site5	CCATGCCCCAGGGCTGGAATG	CACC G CCATGCCCCAGGGCTGGAATG
		TAAC CATTCCAGCCCTGGGGCATGGC
Pcsk9-site6	CCTGCTGCCATGCCCCAGGGC	CACC G CCTGCTGCCATGCCCCAGGGC
		TAAC GCCCTGGGGCATGGCAGCAGGC
HEK1-site1	ATGTGTGTATATGATGATGTG	CACC G ATGTGTGTATATGATGATGTG
		TAAC CACATCATCATATACACACATC
HEK1-site2	CACATCATCATATACACACAT	CACC G CACATCATCATATACACACAT

		TAAC ATGTGTGTATATGATGATGTGC
HEK1-site3	TGGAGCCATCTCACTTCTATC	CACC G TGGAGCCATCTCACTTCTATC
		TAAC GATAGAAGTGAGATGGCTCCAC
HEK1-site4	GGAATACATTTCTTTGGGTCA	CACC G GGAATACATTTCTTTGGGTCA
		TAAC TGACCCAAAGAAATGTATTCCC
HEK1-site5	TAATTAGAGCATAAATAAGAA	CACC G TAATTAGAGCATAAATAAGAA
		TAAC TTCTTATTTATGCTCTAATTAC

Supplementary Table S7. SpCas9 target site sequences in this study.

sgRNA	Target site sequences (5'-3')	oligos
Tyr-site1	AGAAAAGAACTTATTCTTTT	CACC G AGAAAAGAACTTATTCTTTT
		AAAC AAAAGAATAAGTTCTTTTCTC
Tyr-site2	ATGTTGATATCATTAAACAT	CACC G ATGTTGATATCATTAAACAT
		AAAC ATGTTTAATGATATCAACATC
Tyr-site3	ATTTTGCCCATGAAGCACCA	CACC G ATTTTGCCCATGAAGCACCA
		AAAC TGGTGCTTCATGGGCAAAATC
Klkb1-site1	CCACACTGCTTTAAAGAATG	CACC G CCACACTGCTTTAAAGAATG
		AAAC CATTCTTTAAAGCAGTGTGGC
Klkb1-site2	GGTCTTAGAGATATTAAAGT	CACC G GGTCTTAGAGATATTAAAGT
		AAAC ACTTTAATATCTCTAAGACCC
Klkb1-site3	GCACTTCTTGATTGCTTCAT	CACC G GCACTTCTTGATTGCTTCAT
		AAAC ATGAAGCAATCAAGAAGTGCC
Hpd-site1	GACGTGGCTGACTACCTCCC	CACC G GACGTGGCTGACTACCTCCC
		AAAC GGGAGGTAGTCAGCCACGTCC
Hpd-site2	CTTCTCCACCAGGGTGTGTG	CACC G CTTCTCCACCAGGGTGTGTG
		AAAC CACACACCCTGGTGGAGAAGC
Hpd-site3	TTTGATGGATTCCTCGTAGT	CACC G TTTGATGGATTCCTCGTAGT
		AAAC ACTACGAGGAATCCATCAAAC
Hpd-site4	CTCGTTGATGGGCATTTTGA	CACC G CTCGTTGATGGGCATTTTGA
		AAAC TCAAAATGCCCATCAACGAGC
Pcsk9-site1	CATAGCCCCGGCGGGCAGCC	CACC G CATAGCCCCGGCGGGCAGCC
		AAAC GGCTGCCCGCCGGGGCTATGC
Pcsk9-site2	GCGAGCAAGTGTGACAGCCA	CACC G GCGAGCAAGTGTGACAGCCA
		AAAC TGGCTGTCACACTTGCTCGCC
Pcsk9-site3	GACGTCTTTGGTAGAGAAGT	CACC G GACGTCTTTGGTAGAGAAGT
		AAAC ACTTCTCTACCAAAGACGTCC
Pcsk9-site4	TGCATTCCAGCCCTGGGGCA	CACC G TGCATTCCAGCCCTGGGGCA
		AAAC TGCCCCAGGGCTGGAATGCAC
HEK1-site1	GTTTGATTTTACACATCATCAT	CACC G GTTTGATTTTACACATCATCAT
		AAAC ATGATGATGTGTAAAATCAAACC
HEK1-site2	CTGTTTGATTTTACACATCATC	CACC G CTGTTTGATTTTACACATCATC
		AAAC GATGATGTGTAAAATCAAACAGC

HEK1-site3	TCTATCAATAATTGCTCTGGGT	CACC G TCTATCAATAATTGCTCTGGGT
		AAAC ACCCAGAGCAATTATTGATAGAC
HEK1-site4	ACTACTATCATTTTGCTATAGA	CACC G ACTACTATCATTTTGCTATAGA
		AAAC TCTATAGCAAAATGATAGTAGTC
HEK1-site5	GAAATTTAAAAACTGTAGTGAT	CACC G GAAATTTAAAAACTGTAGTGAT
		AAAC ATCACTACAGTTTTTAAATTTCC

Supplementary sequences

>ISDra2 TnpB
MIRNKAFVVRLYPNAAQTELINRTLGSARFVYNHFLARRIAAYKESGKGLTYG
QTSSELTLLKQAEETSWLSEVDKFALQNSLKNLETAYKNFFRTVKQSGKKVG
FPRFRKKRTGESYRTQFTNNNIQIGEGRLKLPKLGWVKTKGQQDIQGKILNV
TVRRIHEGHYEASVLCEVEIPYLPAAPKFAAGVDVGIKDFAIVTDGVRFKHEQ
NPKYYRSTLKRLRKAQQTLSRRKKGSARYGKAKTKLARIHKRIVNKRQDFLH
KLTTSLVREYEIIGTEHLKPDNMRKNRRLALSISDAGWGEFIRQLEYKAAWYG
RLVSKVSPYFPSSQLCHDCGFKNPEVKNLAVRTWTCPNCGETHDRDENAA
LNIRREALVAAGISDTLNAHGGYVRPASAGNGLRSENHATLVV

>ωRNA:
GATTCAAGAATCCCGAAGTGAAGAATCTTGCCGTCCGTACATGGACTTGC
CCGAACTGTGGGGAAACCCATGACCGAGACGAGAACGCTGCGCTGAAC
ATTCGGCGTGAAGCGTTGGTGGCTGCGGGAATCTCAGACACCTTAAACG
CTCATGGAGGCTATGTCAGACCTGCTTCGGCGGGCAATGGTCTGCGAAG
TGAGAATCACGCGACTTTAGTCGTGTGAGGTTCAA

>ωRNA -v2(ωRNA*):
tGGTGGCTGCGGGAATCTCAGACACCTTAAACGCTCATGGAGGCTATgaaa
ATGGTCTGCGAAGTGAGAATCACGCGACTTTAGTCGTGTGAGGTTCAA

>All-in-one TnpB-ωRNA* plasmid
(TnpB highlighted in red and ωRNA* in blue, the plasmid was deposited in benchling (https://benchling.com/s/seq-0v72EZWTPKkMWi9XaljG?m=slm-w6LcjpDFmqrVsYzeJylJ))
TATCCCCTGATTCTCTCGAGTTAAccctagaaagataatcatattgtgacgtacgttaaagat
aatcatgcgtaaaattgacgcatgtgttttatcgAtctgtatatcgaggtttatttattaatttgaatagatattaagt
tttattatatttacacttacatactaataataaattcaacaaacaatttatttatgtttatttatttattaaaaaaaaac
aaaaactcaaaatttcttctataaagtaacaaaacttttaaacattctctcttttacaaaaataaacttattttgtac
tttaaaaacagtcatgttgtattataaaataagtaattagcttaacttatacataatagaaacaaattatacttatt
aattaATCTAGAggtacccgttacataacttacggtaaatggcccgcctggctgaccgcccaacgaccc
ccgcccattgacgtcaatagtaacgccaatagggactttccattgacgtcaatgggtggagtatttacggtaa
actgcccacttggcagtacatcaagtgtatcatatgccaagtacgccccctattgacgtcaatgacggtaaat
ggcccgcctggcattGtgcccagtacatgaccttatgggactttcctacttggcagtacatctacgtattagtc
atcgctattaccatggtcgaggtgagccccacgttctgcttcactctccccatctcccccccctccccaccccc
aattttgtatttatttattttttaattattttgtgcagcgatgggggcggggggggggggggggcgcgcgccagg
cggggcggggcggggcgaggggcggggcggggcgaggcggagaggtgcggcggcagccaatcag
agcggcgcgctccgaaagtttccttttatggcgaggcggcggcggcggcggccctataaaaagcgaagc
gcgcggcgggcgggagtcgctgcgacgctgccttcgccccgtgccccgctccgccgccgcctcgcgccg
cccgccccggctctgactgaccgcgttactcccacaggtgagcgggcgggacggcccttctcctccgggct
gtaattagctgagcaagaggtaagggtttaagggatggttggttggtggggtattaatgtttaattacctggag
cacctgcctgaaatcactttttttcaggttGGGAAATtaatacgactcactataggGaccggtGCGGC
CGCgccaccATGggatcTCCAAAAAAGAAACGGAAGGTGGGATCtATGATCCG

GAACAAGGCCTTCGTGGTGAGGCTGTACCCCAACGCCGCCCAGACAGA
GCTGATCAATAGAACCCTGGGCAGCGCCAGGTTCGTGTACAATCACTTCC
TGGCCAGGAGGATCGCCGCCTACAAGGAGTCCGGCAAGGGCCTGACAT
ACGGCCAGACATCCAGCGAGCTGACCCTGCTGAAGCAGGCCGAGGAGA
CATCCTGGCTGAGCGAGGTGGACAAGTTTGCCCTGCAGAACAGCCTGAA
GAATCTGGAGACCGCCTACAAGAACTTCTTCAGAACAGTGAAGCAGAGC
GGCAAGAAGGTGGGCTTTCCCAGGTTCAGAAAGAAGAGAACAGGCGAG
AGCTACAGGACACAGTTTACAAATAACAACATCCAGATCGGCGAGGGCAG
GCTGAAGCTGCCTAAGCTGGGCTGGGTtAAGACAAAGGGCCAGCAGGAT
ATTCAGGGCAAGATCCTGAATGTGACAGTGAGAAGGATTCATGAGGGCC
ACTACGAGGCCAGCGTGCTGTGTGAGGTGGAGATCCCCTACCTGCCCG
CCGCTCCTAAGTTCGCCGCCGGAGTTGATGTGGGCATCAAGGACTTTGC
CATCGTGACCGACGGCGTGAGATTTAAGCACGAGCAGAACCCTAAGTAC
TACAGGAGCACACTGAAGAGGCTGAGGAAGGCCCAGCAGACACTGTCC
AGAAGAAAGAAGGGCAGCGCCCGGTACGGCAAGGCCAAGACAAAGCTG
GCCAGGATTCATAAGAGAATCGTGAATAAGAGGCAGGACTTTCTGCACAA
GCTGACCACAAGCCTGGTGAGGGAGTACGAGATCATCGGCACAGAGCA
CCTGAAGCCTGATAACATGAGGAAGAACAGgAGACTGGCCCTGTCCATCA
GCGATGCCGGCTGGGGAGAGTTTATCAGACAGCTGGAGTACAAGGCCG
CCTGGTACGGCAGACTGGTGAGCAAGGTGAGCCCTTACTTCCCCAGCTC
CCAGCTGTGTCACGACTGCGGCTTCAAGAATCCCGAGGTGAAGAACCTG
GCCGTGAGAACCTGGACCTGTCCCAACTGCGGCGAGACCCACGACAGA
GATGAAAATGCCGCCCTGAACATCAGGAGGGAGGCCCTGGTGGCCGCC
GGAATTAGCGACACACTGAATGCCCACGGCGGCTACGTGAGACCTGCCT
CTGCCGGAAATGGCCTGAGGTCCGAGAACCACGCCACCCTGGTGGTGG
GtTCCaaaaggccggcggccacgaaaaaggccggccaggcaaaaaagaaaaagggatcTGAT
TACAAAGACGATGACGATAAGGAATTCggtagtgggGAGGGCAGAGGAAGTC
TGCTAACATGCGGTGACGTCGAGGAGAATCCTGGCCCAatggtgagcaagggc
gaggaggataacatggccatcatcaaggagttcatgcgcttcaaggtgcacatggagggctccgtgaacg
gccacgagttcgagatcgagggcgagggcgagggccgcccctacgagggcacccagaccgccaagct
gaaggtgaccaagggtggcccccctgcccttcgcctgggacatcctgtcccctcagttcatgtacggctccaa
ggcctacgtgaagcaccccgccgacatccccgactacttgaagctgtccttccccgagggcttcaagtggg
agcgcgtgatgaacttcgaTgacggcggcgtggtgaccgtgacccaggactcctccctgcaggacggcg
agttcatctacaaggtgaagctgcgcggcaccaacttcccctccgacggccccgtaatgcagaagaaAa
ccatgggctgggaggcctcctccgagcggatgtaccccgaggacggcgccctgaagggcgagatcaag
cagaggctgaagctgaaggacggcggccactacgacgctgaggtcaagaccacctacaaggccaaga
agcccgtgcagctgcccggcgcctacaacgtcaacatcaagttggacatcacctcccacaacgaggact
acaccatcgtggaacagtacgaacgcgccgagggccgccactccaccggcggcatggacgagctgtac
aagTGAAAGCTTAACTTGTTTATTGCAGCTTATAATGGTTACAAATAAAGCAA
TAGCATCACAAATTTCACAAATAAAGCATTTTTTTCACTGCATTCTAGTTGT
GGTTTGTCCAAACTCATCAATGTATCTTATCATGTCTGGATCACTAGTgaggg
cctatttcccatgattccttcatatttgcatatacgatacaaggctgttagagagataattggaattaatttgactgt
aaacacaaagatattagtacaaaatacgtgacgtagaaagtaataatttcttgggtagtttgcagttttaaaat
tatgttttaaaatggactatcatatgcttaccgtaacttgaaagtatttcgatttcttggctttatatatcttGTGGA

AAGGACGAAACACCGtGGTGGCTGCGGGAATCTCAGACACCTTAAACGC
TCATGGAGGCTATgaaaATGGTCTGCGAAGTGAGAATCACGCGACTTTAGT
CGTGTGAGGTTCAAATGTCTTCCTGGGACGAAGACAAggccggcatggtcccag
cctcctcgctggcgccggctgggcaacatgcttcggcatggcgaatgggacTTTTTTTacgcgtTaggt
cttgaaaggagtgggaattggctccggtgcccgtcagtgggcagagcgcacatcgcccacagtccccga
gaagttggggggaggggtcggcaattgatccggtgcctagagaaggtggcgcggggtaaactgggaaa
gtgatgtcgtgtactggctccgcctttttcccgagggtgggggagaaccgtatataagtgcagtagtcgccgt
gaacgttctttttcgcaacgggtttgccgccagaacacaggacGCTAGcgccaccatgagccggctgg
acaagagcaaagtgatcaacagcgccctggaactgctgaacggcgtgggcatcgagggcctgaccacc
cggaagctggcccagaaactgggcgtggaacagcccaccctgtactggcacgtgaagaacaagcggg
ccctgctggacgccctgcccatcgagatgctggaccggcaccacacccacagctgccctctggaaggcg
agagctggcaggacttcctgcggaacaacgccaagagctacagatgcgccctgctgagccaccgggac
ggcgccaaagtgcacctgggcaccagacccaccgagaagcagtacgagacactggaaaaccagctg
gccttcctgtgccagcagggcttcagcctggaaaacgccctgtacgccctgagcgccgtgggccacttcac
cctgggctgcgtgctggaagaacaggaacaccaggtcgccaaagaggaaagagagacacccaccac
cgacagcatgccccccctgctgaagcaggccatcgagctgttcgacagacagggcgccgagcccgcctt
cctgttcggcctggaactgatcatctgcggcctcgagaagcagctgaagtgcgagagcggcggacccac
cgacgccctggacgacttcgacctggacatgctgcccgccgatgccctggatgattttgatctggatatgctc
cctgctgacgctctcgatgacttcgatctcgatatgctgccaggcggtagtgggGCAACAAACTTCT
CTCTGCTGAAACAAGCCGGAGATGTCGAAGAGAATCCTGGACCGACCGA
GTACAAGCCCACGGTGCGCCTCGCCACCCGCGACGACGTCCCCAGGGC
CGTACGCACCCTCGCCGCCGCGTTCGCCGACTACCCCGCCACGCGCCA
CACCGTCGATCCGGACCGCCACATCGAGCGGGTCACCGAGCTGCAAGA
ACTCTTCCTCACGCGCGTCGGGCTCGACATCGGCAAGGTGTGGGTCGC
GGACGACGGCGCCGCGGTGGCGGTCTGGACCACGCCGGAGAGCGTCG
AAGCGGGGGCGGTGTTCGCCGAGATCGGCCCGCGCATGGCCGAGTTGA
GCGGTTCCCGGCTGGCCGCGCAGCAACAGATGGAAGGCCTCCTGGCGC
CGCACCGGCCCAAGGAGCCCGCGTGGTTCCTGGCCACCGTCGGAGTCT
CGCCCGACCACCAGGGCAAGGGTCTGGGCAGCGCCGTCGTGCTCCCC
GGAGTGGAGGCGGCCGAGCGCGCCGGGGTGCCCGCCTTCCTGGAGAC
CTCCGCGCCCCGCAACCTCCCCTTCTACGAGCGGCTCGGCTTCACCGTC
ACCGCCGACGTCGAGGTGCCCGAAGGACCGCGCACCTGGTGCATGACC
CGCAAGCCCGGTGCCtgaCTTAAGctgtgccttctagttgccagccatctgttgtttgcccctccc
ccgtgccttccttgaccctggaaggtgccactcccactgtcctttcctaataaaatgaggaaattgcatcgcatt
gtctgagtaggtgtcattctattctggggggtggggtggggcaggacagcaagggggaggattgggaaAa
caatagcaggcatgctggggatgcggtgggctctatgggtcgacGGCGCGCCCTAGACGAAG
CAGCTCCAGCCTACACAATCGCTCAAGACGTGTAATGCTtttattatatattagtcac
gatatctataacaagaaaatatatatataataagttatcacgtaagtagaacatgaaataacaatataattat
cgtatgagttaaatcttaaaagtcacgtaaaagataatcatgcgtcattttgactcacgcggtcgttatagttca
aaatcagtgacacttaccgcattgacaagcacgcctcacgggagctccaagcggcgactgagatgtccta
aatgcacagcgacggattcgcgctatttagaaagagagagcaatatttcaagaatgcatgcgtcaattttac
gcagactatctttctagggTTAAGGATCCGCACTTTTCGGGGAAATGTGCGCGGAA
CCCCTATTTGTTTATTTTTCTAAATACATTCAAATATGTATCCGCTCATGAGA
CAATAACCCTGATAAATGCTTCAATAATATTGAAAAAGGAAGAGTATGAGTA

TTCAACATTTCCGTGTCGCCCTTATTCCCTTTTTTGCGGCATTTTGCCTTC
CTGTTTTTGCTCACCCAGAAACGCTGGTGAAAGTAAAAGATGCTGAAGAT
CAGTTGGGTGCACGAGTGGGTTACATCGAACTGGATCTCAACAGCGGTA
AGATCCTTGAGAGTTTTCGCCCCGAAGAACGTTTTCCAATGATGAGCACT
TTTAAAGTTCTGCTATGTGGCGCGGTATTATCCCGTATTGACGCCGGGCA
AGAGCAACTCGGTCGCCGCATACACTATTCTCAGAATGACTTGGTTGAGT
ACTCACCAGTCACAGAAAAGCATCTTACGGATGGCATGACAGTAAGAGAA
TTATGCAGTGCTGCCATAACCATGAGTGATAACACTGCGGCCAACTTACTT
CTGACAACGATCGGAGGACCGAAGGAGCTAACCGCTTTTTTGCACAACA
TGGGGGATCATGTAACTCGCCTTGATCGTTGGGAACCGGAGCTGAATGA
AGCCATACCAAACGACGAGCGTGACACCACGATGCCTGTAGCAATGGCA
ACAACGTTGCGCAAACTATTAACTGGCGAACTACTTACTCTAGCTTCCCG
GCAACAATTAATAGACTGGATGGAGGCGGATAAAGTTGCAGGACCACTTC
TGCGCTCGGCCCTTCCGGCTGGCTGGTTTATTGCTGATAAATCTGGAGCC
GGTGAGCGTGGGTCTCGCGGTATCATTGCAGCACTGGGGCCAGATGGTA
AGCCCTCCCGTATCGTAGTTATCTACACGACGGGGAGTCAGGCAACTATG
GATGAACGAAATAGACAGATCGCTGAGATAGGTGCCTCACTGATTAAGCA
TTGGTAACTGTCAGACCAAGTTTACTCATATATACTTTAGATTGATTTAAAAC
TTCATTTTTAATTTAAAAGGATCTAGGTGAAGATCCTTTTTGATAATCTCATG
ACCAAAATCCCTTAACGTGAGTTTTCGTTCCACTGAGCGTCAGACCCCGT
AGAAAAGATCAAAGGATCTTCTTGAGATCCTTTTTTTCTGCGCGTAATCTG
CTGCTTGCAAACAAAAAAACCACCGCTACCAGCGGTGGTTTGTTTGCCG
GATCAAGAGCTACCAACTCTTTTTCCGAAGGTAACTGGCTTCAGCAGAGC
GCAGATACCAAATACTGTTCTTCTAGTGTAGCCGTAGTTAGGCCACCACTT
CAAGAACTCTGTAGCACCGCCTACATACCTCGCTCTGCTAATCCTGTTACC
AGTGGCTGCTGCCAGTGGCGATAAGTCGTGTCTTACCGGGTTGGACTCA
AGACGATAGTTACCGGATAAGGCGCAGCGGTCGGGCTGAACGGGGGGT
TCGTGCACACAGCCCAGCTTGGAGCGAACGACCTACACCGAACTGAGAT
ACCTACAGCGTGAGCTATGAGAAAGCGCCACGCTTCCCGAAGGGAGAAA
GGCGGACAGGTATCCGGTAAGCGGCAGGGTCGGAACAGGAGAGCGCAC
GAGGGAGCTTCCAGGGGGAAACGCCTGGTATCTTTATAGTCCTGTCGGG
TTTCGCCACCTCTGACTTGAGCGTCGATTTTTGTGATGCTCGTCAGGGGG
GCGGAGCCTATGGAAAAACGCCAGCAACGCGGCCTTTTTACGGTTCCTG
GCCTTTTGCTGGCCTTTTGCTCACATGTTCTTTCCTGCGT

1 Clement, K. *et al.* CRISPResso2 provides accurate and rapid genome editing sequence analysis. *Nature biotechnology* **37**, 224-226, doi:10.1038/s41587-019-0032-3 (2019).

2 Yin, J. *et al.* Optimizing genome editing strategy by primer-extension-mediated sequencing. *Cell discovery* **5**, 18, doi:10.1038/s41421-019-0088-8 (2019).

3 Charlesworth, C. T. *et al.* Identification of preexisting adaptive immunity to Cas9 proteins in humans. *Nature medicine* **25**, 249-254, doi:10.1038/s41591-018-0326-x (2019).

4 Wang, D. *et al.* Adenovirus-Mediated Somatic Genome Editing of Pten by CRISPR/Cas9 in Mouse Liver in Spite of Cas9-Specific Immune Responses. *Human gene therapy* **26**, 432-442, doi:10.1089/hum.2015.087 (2015).
