# Supplementary material for: Engineering a transposon-associated TnpB-ωRNA system for efficient gene editing and phenotypic correction of a tyrosinaemia mouse model
Source: Nat Commun. 2024 Jan 27;15:831. doi: 10.1038/s41467-024-45197-z (PMC10821889; doi:10.1038/s41467-024-45197-z)
Supplement: Supplementary file 3 — Reporting Summary [file 41467_2024_45197_MOESM3_ESM.pdf]

nature portfolio

Corresponding author(s): Chunlong Xu, Yingsi Zhou, Chunyi Hu

Last updated by author(s): Dec 31, 2023

Reporting Summary

Nature Portfolio wishes to improve the reproducibility of the work that we publish. This form provides structure for consistency and transparency in reporting. For further information on Nature Portfolio policies, see our Editorial Policies and the Editorial Policy Checklist.

Statistics

For all statistical analyses, confirm that the following items are present in the figure legend, table legend, main text, or Methods section.

n/a	Confirmed	
☐	☒	The exact sample size (*n*) for each experimental group/condition, given as a discrete number and unit of measurement
☐	☒	A statement on whether measurements were taken from distinct samples or whether the same sample was measured repeatedly
☐	☒	The statistical test(s) used AND whether they are one- or two-sided<br>*Only common tests should be described solely by name; describe more complex techniques in the Methods section.*
☒	☐	A description of all covariates tested
☒	☐	A description of any assumptions or corrections, such as tests of normality and adjustment for multiple comparisons
☐	☒	A full description of the statistical parameters including central tendency (e.g. means) or other basic estimates (e.g. regression coefficient) AND variation (e.g. standard deviation) or associated estimates of uncertainty (e.g. confidence intervals)
☐	☒	For null hypothesis testing, the test statistic (e.g. *F*, *t*, *r*) with confidence intervals, effect sizes, degrees of freedom and *P* value noted<br>*Give P values as exact values whenever suitable.*
☒	☐	For Bayesian analysis, information on the choice of priors and Markov chain Monte Carlo settings
☒	☐	For hierarchical and complex designs, identification of the appropriate level for tests and full reporting of outcomes
☒	☐	Estimates of effect sizes (e.g. Cohen's *d*, Pearson's *r*), indicating how they were calculated

Our web collection on statistics for biologists contains articles on many of the points above.

Software and code

Policy information about availability of computer code

Data collection	There is no public data collected in the study.
Data analysis	Flow cytometry data was analyzed by Flowjo V10. Statistics analysis and plotting were performed with Prism 9. Deep-seq data was analyzed using CRISPResso2. PEM-seq analysis was performed following the pipeline described previously in Github (https://github.com/liumz93/PEM-Q).

For manuscripts utilizing custom algorithms or software that are central to the research but not yet described in published literature, software must be made available to editors and reviewers. We strongly encourage code deposition in a community repository (e.g. GitHub). See the Nature Portfolio guidelines for submitting code & software for further information.

Data

Policy information about availability of data

All manuscripts must include a data availability statement. This statement should provide the following information, where applicable:

- Accession codes, unique identifiers, or web links for publicly available datasets
- A description of any restrictions on data availability
- For clinical datasets or third party data, please ensure that the statement adheres to our policy

Deep-seq data is deposited to the GEO repository under accession number PRJNA973546.

Research involving human participants, their data, or biological material

Policy information about studies with human participants or human data. See also policy information about sex, gender (identity/presentation), and sexual orientation and race, ethnicity and racism.

Reporting on sex and gender	Since we need to investigate the prevalence of anti-TnpB or SpCas9 immunity in human population, sex and gender of blood sample were not used in the study as criteria for sample collection.
Reporting on race, ethnicity, or other socially relevant groupings	No race, ethnicity or socially relevant groupings were performed in our study.
Population characteristics	No recruitment criteria was used and demographic information about our blood samples were also de-identified for the patients privacy.
Recruitment	De-identified blood samples were obtained with the patient consent in strict observance of the legal and institutional ethical regulations. No recruitment criteria was used and demographic information about our blood samples were also de-identified for the patient privacy.
Ethics oversight	Institutional Review board of International Peace Maternity and Child Health Hospital, School of Medicine, Shanghai Jiao Tong University, Shanghai, China.

Note that full information on the approval of the study protocol must also be provided in the manuscript.

Field-specific reporting

Please select the one below that is the best fit for your research. If you are not sure, read the appropriate sections before making your selection.

☒ Life sciences ☐ Behavioural & social sciences ☐ Ecological, evolutionary & environmental sciences

For a reference copy of the document with all sections, see nature.com/documents/nr-reporting-summary-flat.pdf

Life sciences study design

All studies must disclose on these points even when the disclosure is negative.

Sample size	No sample size calculation was performed in this study. Sample sizes were chosen after deep sequencing depending on the read number and quality. All sample sizes were sufficient for the following statistical test.
Data exclusions	No data was excluded.
Replication	We tested experimental conditions using different gRNAs to ensure robustness. For all experiments performed in the study, three biologically independent replicates were performed, unless otherwise noted.
Randomization	Randomization was used in all experiments.
Blinding	No blinding was applied, due to no subjective assessments were required.

Reporting for specific materials, systems and methods

We require information from authors about some types of materials, experimental systems and methods used in many studies. Here, indicate whether each material, system or method listed is relevant to your study. If you are not sure if a list item applies to your research, read the appropriate section before selecting a response.

Materials & experimental systems

n/a	Involved in the study	
☐	☒	Antibodies
☐	☒	Eukaryotic cell lines
☒	☐	Palaeontology and archaeology
☐	☒	Animals and other organisms
☒	☐	Clinical data
☒	☐	Dual use research of concern
☒	☐	Plants

Methods

n/a	Involved in the study	
☒	☐	ChIP-seq
☐	☒	Flow cytometry
☒	☐	MRI-based neuroimaging

Antibodies

Antibodies used	Anti-CRISPR-Cas9(ET1703, H UABIO) ,Anti-Human-lgG(ALPVHHs),Anti-dstrophin(Abcam, ab15277). anti-Vinculin(Cell Signaling Technology, 13901S).anti-HPD(SantaCruz, sc-390279), anti-P21(Abcam, ab109199),anti-laminin-2(Sigma-Aldrich, L0663)
Validation	Anti-CRISPR-Cas9(ET1703, HUABIO;dilution 1:1000), Anti-Human-lgG(ALPVHHs,dilution 1:1000), anti-HPD antibody(SantaCruz, sc-390279; dilution 1:100 or 1:500), anti-P21 antibody (Abcam, ab109199; dilution 1:200), anti-Vinculin(Cell Signaling Technology, 13901S; dilution 1:100), anti-dystrophin antibody (Abcam, ab15277; dilution 1:100), anti-laminin-2 antibody (Sigma-Aldrich, L0663; dilution 1:100)

Eukaryotic cell lines

Policy information about cell lines and Sex and Gender in Research

Cell line source(s)	HEK293T and N2a cells were purchased from Stem Cell Bank, Chinese Academy of Sciences.
Authentication	None of the cell lines were authenticated by us.
Mycoplasma contamination	All cell lines tested negative for mycoplasma contamination by PCR.
Commonly misidentified lines (See ICLAC register)	None misidentified lines listed in the database of ICLAC were used.

Animals and other research organisms

Policy information about studies involving animals; ARRIVE guidelines recommended for reporting animal research, and Sex and Gender in Research

Laboratory animals	The Fah-/- mouse model harbors the same homozygous G to A point mutation of the last nucleotide of exon 8, which result in exon skipping and the loss of FAH. The Fah-/- mice mice were kept on 10 mg/L NTBC water. Fah -/- mice: femal and male, 8 week-old.<br>B6D2F1(C57BL/6J X DBA2) mice: female, 8 week-old.<br>ICR mice: females, 8 week-old.<br>C57BL/6J mice: 8 week-old.
Wild animals	No wild animals were used in this study.
Reporting on sex	Both male and female mice were collected for this study.
Field-collected samples	This study did not involve samples collected from the field.
Ethics oversight	All animal experiments were performed and approved by the Animal Care and Use Committee of Shanghai Center for Brain Science and Brain-Inspired Technology, Lingang Laboratory, Shanghai, China.

Note that full information on the approval of the study protocol must also be provided in the manuscript.

Flow Cytometry

Plots

Confirm that:

☒ The axis labels state the marker and fluorochrome used (e.g. CD4-FITC).

☒ The axis scales are clearly visible. Include numbers along axes only for bottom left plot of group (a 'group' is an analysis of identical markers).

☒ All plots are contour plots with outliers or pseudocolor plots.

☒ A numerical value for number of cells or percentage (with statistics) is provided.

Methodology

Sample preparation	Cells were digested by 0.05% trypsin, resuspended with cell culture medium, and analyzed by flow cytometry.
Instrument	BD FACSAria III
Software	Flowjo V10
Cell population abundance	Cell population abundance was influenced by the size of the plasmids.

Gating strategy

Positive and negative boundaries were determined by control cells that were not transfected with any plasmids.

☒ Tick this box to confirm that a figure exemplifying the gating strategy is provided in the Supplementary Information.
